# Comparative Whole Cell Proteomics of *Listeria monocytogenes* at Different Growth Temperatures

**DOI:** 10.4014/jmb.1911.11027

**Published:** 2019-12-02

**Authors:** Soyoon Won, Jeongmin Lee, Jieun Kim, Hyungseok Choi, Jaehan Kim

**Affiliations:** Department of Food and Nutrition, Chungnam National University, Daejeon 305-764, Republic of Korea

**Keywords:** *Listeria monocytogenes*, bacterial cell growth, temperature, protein expression, shotgun proteomics, bacterial proteomics

## Abstract

*Listeria monocytogenes* is a gram-positive, facultative anaerobe food pathogen responsible for the listeriosis that mostly occurs during the low-temperature storage of a cold cut or dairy products. To understand the systemic response to a wide range of growth temperatures, *L. monocytogenes* were cultivated at a different temperature from 10°C to 42°C, then whole cell proteomic analysis has been performed both exponential and stationary cells. The specific growth rate increased proportionally with the increase in growth temperature. The maximum growth rate was observed at 37°C and was maintained at 42°C. Global protein expression profiles mainly depended on the growth temperatures showing similar clusters between exponential and stationary phases. Expressed proteins were categorized by their belonging metabolic systems and then, evaluated the change of expression level in regard to the growth temperature and stages. DnaK, GroEL, GroES, GrpE, and CspB, which were the heat&cold shock response proteins, increased their expression with increasing the growth temperatures. In particular, GroES and CspB were expressed more than 100-fold than at low temperatures during the exponential phase. Meanwhile, CspL, another cold shock protein, overexpressed at a low temperature then exponentially decreased its expression to 65-folds. Chemotaxis protein CheV and flagella proteins were highly expressed at low temperatures and stationary phases. Housekeeping proteins maintained their expression levels constant regardless of growth temperature or growth phases. Most of the growth related proteins, which include central carbon catabolic enzymes, were highly expressed at 30°C then decreased sharply at high growth temperatures.

## Introduction

*Listeria monocytogenes* is a gram-positive, facultative anaerobic organism and major causative agent of listeriosis [1], a potentially life-threatening illness and menace to public health [1, 2]. Listeriosis is commonly found in the elderly, pregnant women, newborns, and immunocompromised individuals [3-6]. *L. monocytogenes* has been reported to show high resistance to environmental stress compared with other non-sporing, food borne pathogens [7]. One of the major problematic characteristics of *L. monocytogenes* is the ability to grow at a wide range of temperatures (-0.4 to 44°C) [8]. Due to it adaptation to low temperature, *L. monocytogenes* is often found in the food stored in refrigerators such as cold cuts or ice cream [9]. The ability of the bacterium to proliferate under adverse conditions and survive under a stress environment might mediate various mechanisms that enable it to adapt and show maximum survival [10-12].

In addition to the contamination of the cold storage food, *L. monocytogenes* expand its territories to the agricultural products. In September 2011, Cantaloupe was implicated in a multistate outbreak of foodborne listeriosis in the USA and it was the first listeriosis outbreak associated with melon such as fresh-cut products [13]. On this outbreak, 147 cases have been reported that include 33 patients have died and 143 were hospitalized across the 28 states. Since then, listeriosis has been occurred every year in multistate with a high motility rate [14, 15]. In Korea, there are many traditional fermented foods stored at a low temperature. Particularly, kimchi naturally necessitates the low-temperature storage, microbial cell growth and the use of non-sterilized vegetables, all of which could increase the possibility of listeriosis [16, 17].

One of the essential characteristics of *L. monocytogenes* is the endurance of environmental stress such as the change of temperature that commonly occurred during the processing of food. Environmental stress could induce the protection mechanisms or the specific heat shock response of bacterium. Since those unique mechanisms protect the protein/enzyme against the chemical damage or misfolding, the maintenance of the stress response protein expression is crucial for bacterial growth as well as survival against stress conditions [18, 19].

Studies on the complete genome of *L. monocytogenes* have opened the ways to investigate mechanisms involved in the survival of bacteria under stress conditions [20]. Two-dimensional electrophoresis and other biological methods have been employed for the expression of cold shock protein in response to cold temperature [21]. In addition, the simple proteomic approach has been applied to investigate the impact of the different stress conditions such as acid, alkali, heat and salt concentration [22]. Quantitative analysis of protein mixture has been performed via two-dimensional polyacrylamide gel electrophoresis (2D-PAGE) followed by mass spectrometry (MS) or tandem mass spectrometry (MS/MS) [23]. However, the expression of protein has been found to exhibit a strong variation under different conditions. There are a limited number of studies that showed the protein expression profile of *L. monocytogenes* under stress conditions including low and high temperatures as well as growth phases [24-26].

Proteomic studies on the growth of *L. monocytogenes* and the adaptation at refrigeration temperatures were lacking. Further, profiling of global protein expression in *L. monocytogenes* to understand the systemic changes in a microbial metabolic network was still a pioneering area. We, in this study, are focusing on the differential expression of proteins involved in the adaptation at different tempera-tures. In addition, metabolic changes in the stationary phase were studied considering the long term storage in the refrigerator.

## Materials and Methods

### Reagents/Chemicals

All chemicals were purchased from Sigma-Aldrich (USA). Microbiology culture media was obtained from Difco (Becton Dickinson, USA). *Listeria monocytogenes* EGD-e strain (ATCC BAA-679) was obtained from the American Tissue Culture Collection (USA).

### Bacterial Cell Growth

Cell stocks were routinely maintained in Brain Heart Infusion (BHI) media which contains brain heart, infusion from solids 8 g, Peptic Digest of Animal Tissue 5 g, Pancreatic Digest of Casein 16 g, Sodium Chloride 5 g, glucose 2 g, and Disodium Hydrogen Phosphate 2.5 g/l. *L. monocytogenes* was grown on brain heart infusion (BHI) under different temperatures at 10°C, 20°C, 30°C, 30°C, and 42°C, respectively. The growth rate was measured via optical density at 600 nm. All experiments were performed in duplicate.

### Sample Preparation for Proteomic Analysis

*L. monocytogenes* cells were collected at the exponential and stationary phase, respectively. After centrifugation, 50 ml of cell pellets were washed three times with phosphate-buffered saline (PBS) to remove the remaining media. The cell pellet was resuspended in 1 ml of urea buffer (8 M urea buffer/0.1 M Tris HCl). After resuspension, the cell pellet was mechanically disrupted by silica beads in a bead-beater (FastPrep; QBiogene, USA) for eight cycles of 40 sec pulse and 40 sec on ice.

The peptide library for the proteome analysis was prepared as follows. Cell lysates containing 200 mg of proteins were transferred into a new Eppendorf tube, then proteins were precipitated by the addition of ethanol. Three volumes of ethanol (75% (v/v) final) was added on the cell lysates and stored at −20°C for 3 h. Cell lysates were centrifuged at 15,000 ×*g* for 20 min at 4°C. After washing two times, the protein pellet was resuspended in 1ml urea buffer (1 M urea/0.1 M Tris HCl buffer, pH 8.5). For the digestion, 5 µg of mass spectrometry-grade trypsin (Thermo, USA) was added and the reaction was maintained at 37°C overnight. The tryptic peptides were cleaned and concentrated by the Macro Trap with peptide concentration and Desalting cartridge (USA). The resultant peptides were eluted in 90% acetonitrile in water and then dried before analysis by mass spectrometry.

### Mass Spectrometry Analysis

A Nano-LC chip/Q-TOF MS system was used for the analysis of peptides and was equipped with a microwell plate auto sampler (maintained at 4°C), capillary flow pump, nanoflow pump, nano-LC chip/MS interface (Chip Cube) and an Agilent 6540 Q-TOF MS. The chip used a G4240-62001 HPLC-Chip (Agilent Technologies) that incorporated a 40 nL enrichment column and a 75 µm × 43 mm separation column which is packed with Zorbax 300SB-C18 5 µm material.

Peptides were reconstituted in water at concentrations corresponding to between 40 and 200 ng of the original protein per 1 µl injection. The sample was loaded via a capillary pump that delivered 0.1% formic acid in 3.0% acetonitrile/water (v/v) isocratically at 4.0 µl/min. However, sample injection was carried out by nano pump gradient delivered at 0.4 µl/min using (**A**) 0.1% formic acid in 3.0% acetonitrile/water (v/v) and (**B**) 0.1%formic acid in 90.0% acetonitrile/water (v/v). Samples were eluted with 0% B, 0.00–2.0 min; 0 to 5% B, 2.0–30.00 min; 5 to 30%B, 30.00–50.00 min; 30 to 40% B, 50.00–70.00 min; 40 to 50%, 70.00–79.90 min; 50 to 100% and 100 to 0% B, 79.90–80.00 min. This was followed by equilibration at 0% B, 80.00–95.00 min. The drying gas temperature was set at 325°C with a flow rate of 4.0 L/min.

### Database Searching, False Discovery Rate, and Quantitation

Tandem mass spectra were extracted and charge state was deconvoluted by Qualitative Analysis B.04.00. Deisotoping was not performed. All MS/MS datas were analyzed using X!Tandem, which was set up to search against the *L. monocytogenes* whole proteome with a fragment ion mass tolerance of 0.60 Da. Oxidation of methionine was specified as a variable modification. A preliminary protein search was performed using the false discovery rate (FDR) determined by independent searching of MS/MS spectra against the forward (target) and the reversed database (decoy) of *L. monocytogenes*, including plasmid proteins. The FDR was calculated as R/(F+R) where R and F are the number of proteins from the decoy and target database, respectively. Initial protein identification was obtained at a FDR level of 1.5%. Among the proteins obtained, those having a number of unique peptides greater than 2 (P_uniq_ ≥ 2) and a probability score lower than -7 (log(**E**) ≤ -7) were considered to be expressed and were used for further quantitative analysis. The relative quantitation of protein was performed by the spectral counting method [27]. Briefly, a spectral abundance factor of each protein was calculated from the length of a protein (the number of amino acids: *L_k_*) and the number of spectra used to identify the protein (*SpC_k_*). After normalization of each spectral abundance factor in a sample, the value of a normalized spectral abundance factor (NSAF) was used to determine the relative amount of each protein within a sample. For an accurate quantitative calculation, the number of total spectra (*SpC_k_*) of each protein in *L. monocytogenes* had to be greater than 4.

## Results and Discussion

### Impact of Temperature on Cell Growth

**Fig. 1:**
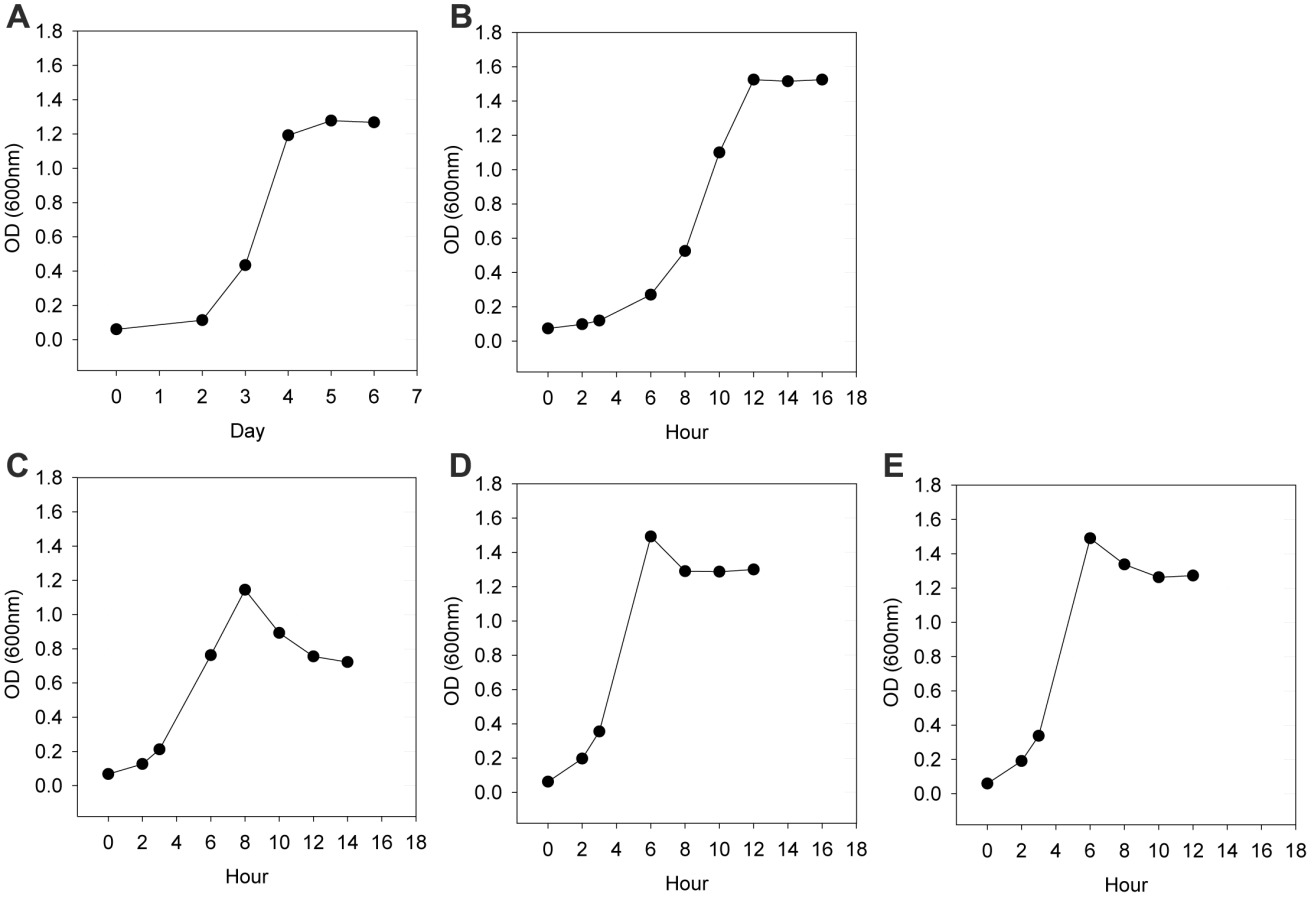
Cell growth profiles of *L. monocytogenes* at (**A**) 10°C, (**B**) 20°C, (**C**) 30°C, (**D**) 37°C, and (**E**) 42°C.

**Fig. 2:**
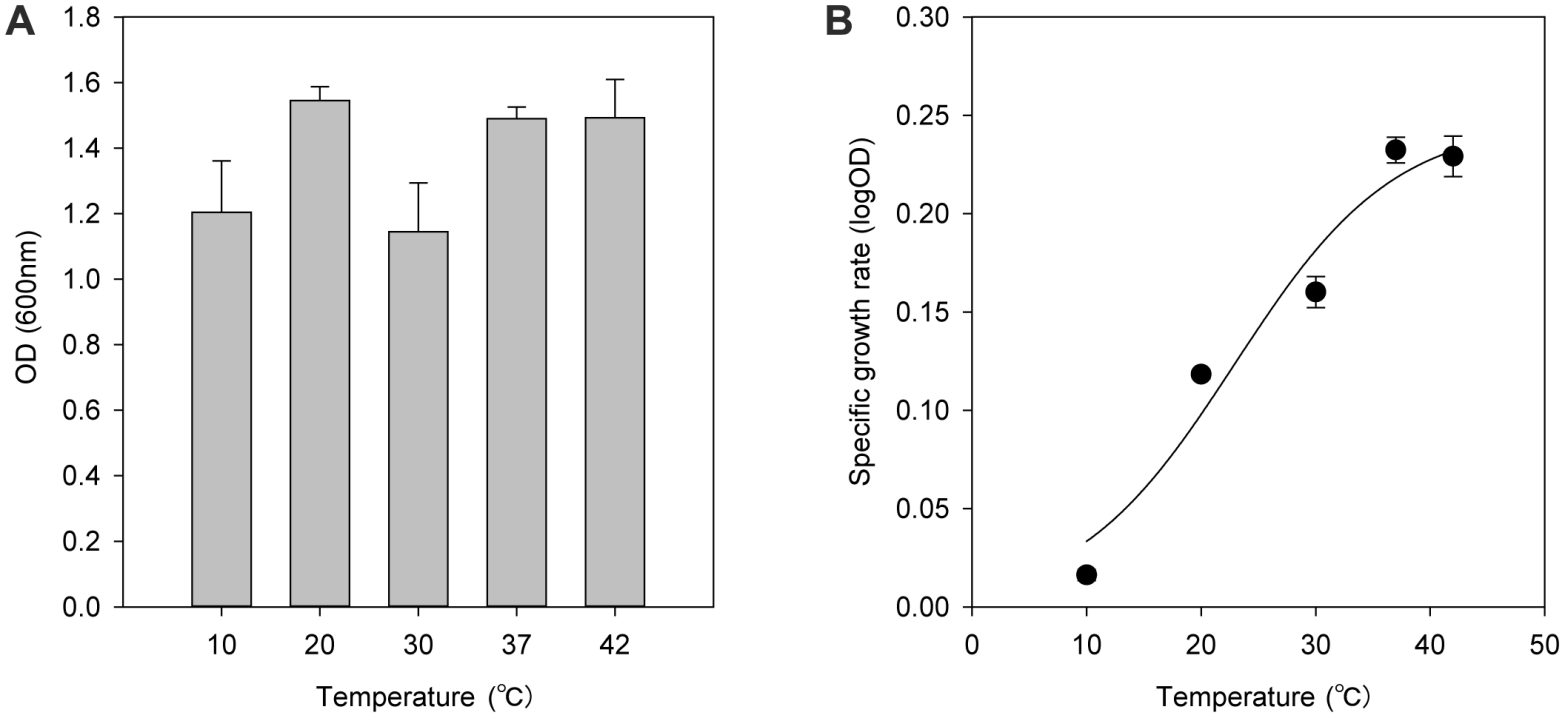
Cell growth kinetic parameters of *L. monocytogenes* at 10°C, 20°C, 30°C, 37°C, and 42°C, (**A**) Maximum cell optical density, (**B**) specific cell growth rates.

*L. monocytogenes* was grown in Brain Heart Infusion (BHI) medium at temperatures of 10, 20, 30, 37, and 42°C (Fig. 1). As shown in Fig. 1, *L. monocytogenes* had a similar growth pattern regardless of the growth temperature. After the exponential phase, *L. monocytogenes* reached the stationary phase with maximum optical density (OD) ranging from 1.20 to 1.54. Maximum cell OD at 10°C and 30°C was slightly lower than other temperatures showing 78% of the maximum OD at 37°C (Fig. 2A). Above 30°C, cell OD was slightly decreased after reaching maximum OD at the stationary phase.

While the maximum cell OD was similar regardless of the temperature, growth rates were proportionally increased to the growth temperature (Fig. 2B). Although the *L. monocytogenes* is able to grow at a low temperature near 0°C, the optical growth temperature was 37°C. At 10°C, the specific cell growth rate was 5% of that observed at 37°C, which makes the cell doubling time more than 2 days. Rather than low temperature adaptation, *L. monocytogenes* maintained its full growth activity at 42°C. It suggested *L. monocytogenes* was adapted not only to the low temperature, but also to a wide range of growth temperatures.

### Protein Expression Profile at Different Temperatures

The total number of MS/MS spectra used to identify the proteins and the number of identified proteins are summarized in Table S1. Approximately 300~420 proteins were identified from each sample of *L. monocytogenes* grown in BHI broth at different temperatures and growth stages. Quantitative expression was assessed after using the normalized spectral abundance factor (NSAF) for each protein. NSAF was found to represent the relative abundance of protein in a sample and was calculated from the total number of spectra used for the identification of protein and the number of amino acids. Between the duplicates, the average CV of NSAF of each protein was less than 18%.

**Fig. 3:**
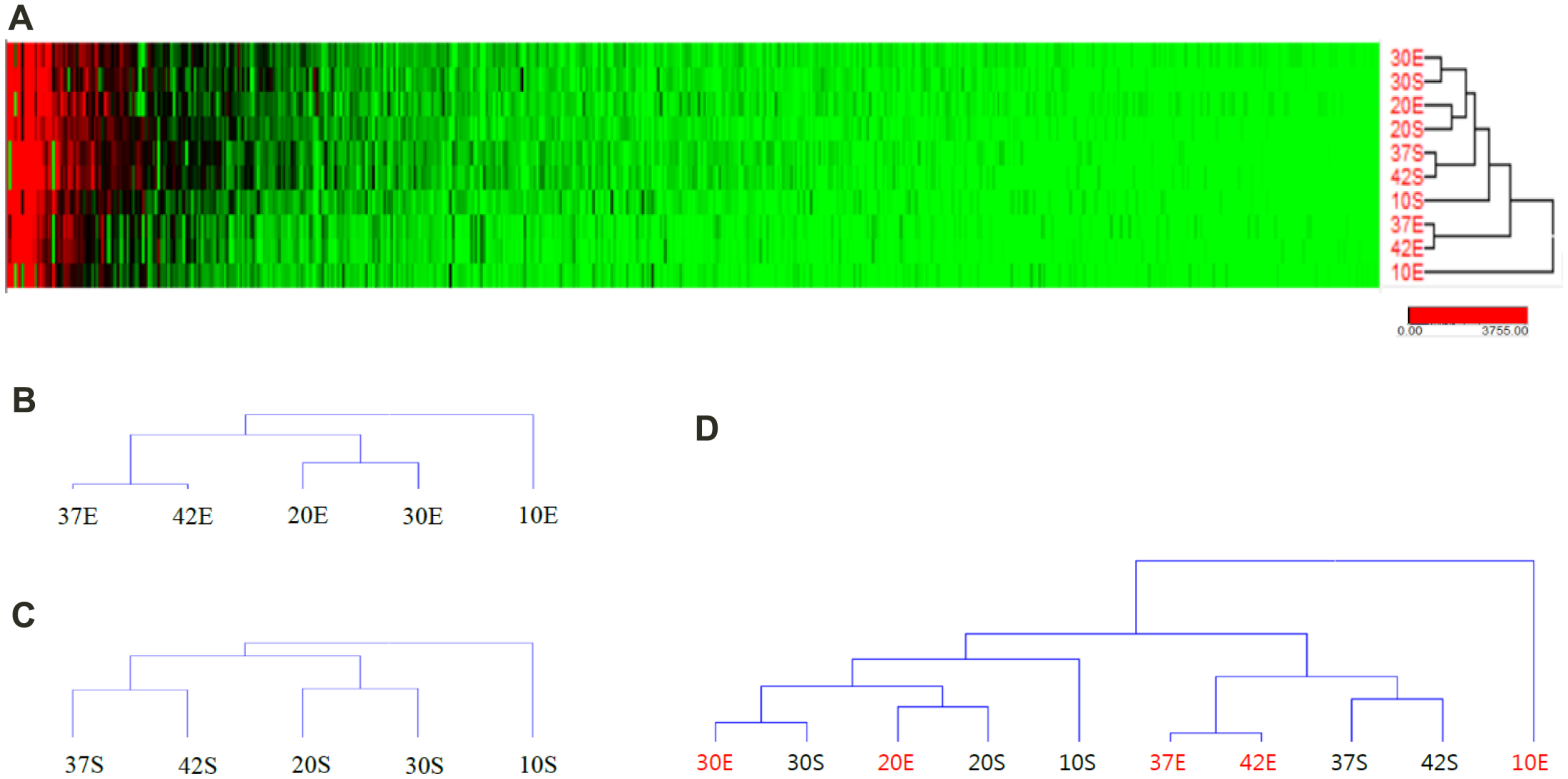
Hierarchical analysis of global protein expression of *L. monocytogenes* at different temperatures and growth phases. Similarity matrix was calculated using Euclidean distance and clusters were constructed by Average Linkage method. Numbers indicated the growth temperature and the E and S meant the exponential and stationary samples, respectively. (**A**) Heatmap, (**B**) the cluster of the exponential samples, (**C**) the cluster of the stationary samples, (**D**) the cluster of all the samples.

The global protein expression profile is shown on a heat map (Fig. 3). The hierarchical cluster analysis was performed to global expression data in different groups based on variables; namely, culture conditions (such as temperature) and growth phase (such as stationary and exponential phase). The similarity matrix was calculated using Euclidean distance from a multiple data alignment resulting in the similarity of the quantitative profile between data.

The global protein expression patterns exhibited interesting points depending on the growth temperature and growth stages. The protein expression patterns mainly depended on the growth temperatures showing an identical pattern of clusters in both exponential and stationary phase samples (Figs. 3B and 3C). Regardless of growth stages, hierarchical cluster analysis suggested that the quantitative protein synthesis between 20°C and 30°C seemed similar, and 37°C and 42°C were also similar to each other. The cellular proteins at 10°C seem to be the most unique.

When the exponential and stationary samples were analyzed all together, the protein expression patterns at 20°C and 30°C between exponential and stationary phases were closely similar resulting in a cluster (Fig. 3D). In contrast, at 10°C, 37°C, and 42°C which are the lower and higher range of temperatures, the protein expression patterns were classified by the growth stages. The exponential phases at three different temperatures (37S, 42S, 10S) were clustered first, then those of the stationary phase (37E, 42E, 10E) were linked separately. Meanwhile, regardless of growth stages, the protein expression profiles at 37°C were similar to those at 42°C rather than other temperatures.

In summary, *L. monocytogenes* maintained global protein expression in the range of 20°C to 30°C when cells were in both exponential or stationary phase. Once the temperature moved away, toward either higher or lower range, the protein expression pattern changed to respond to the outside stress. The cellular responses mainly governed by the cell growth stage caused the segregation of exponential and stationary samples to separate clusters. Meantime, the response of the microbial system to the temperature was able to be clustered accordingly.

### The Expression of the Highly Expressed Protein

Table S2 summarized the highly expressed cellular proteins and their changes in ranks. Ranks were determined by the NSAF which could represent the quantitative portion of each protein among total cellular proteins. The proteins listed in the table were sorted by ranking order of the exponential phase sample grown at 37°C. The actual NSAF values of each protein were summarized in Table S2.

Highly expressed protein can be categorized into several groups. A DNA-binding protein HU, phosphor carrier protein HPr, regulatory protein SpoVG family (RNA-binding), elongation factor-Tu (EF-Tu), EF-Ts, non-heme iron-binding ferritin, and ribosome recycling factor (RRF) were the cell growth-related regulatory proteins (Group 1: Fig. S1) [28-31]. These proteins roughly maintained their rankings regardless of growth temperatures or growth stages. Among over 400 total proteins, their changes in ranks were less than 10%. Only RRF exhibited a drastic drop in rank at 30°C cultivation for both exponential and stationary phase. However, the expression levels of these group 1 proteins exhibited different traits. DNA binding protein-HU, HPr, and RRF showed low level expression at 30°C and increased their expression level when growth temperatures were both increased and decreased. Moreover, this parabolic behavior was clearer in the exponential phase samples (Figs. S1A-S1C). Ferritin, EF-TU, and EF-Ts exhibited the opposite behavior and their expression levels increased from 10°C to 30°C then decreased over 30°C. Also, the expression levels at the stationary phase were higher than those at the exponential phase (Figs. S1D-S1F). Two regulatory proteins (SpoVS family, GI:16802242, 16802243) were stably expressed regardless of growth temperatures or phases.

Interestingly, stress response proteins were highly expressed at 37°C. These are required for the bacterial cell adaptation process [32, 33]. Their expression levels were strongly dependent on the temperatures and growth stages (Fig. S2), For example, cold shock protein (GI: 16804055) and GroES were the 2^nd^ and 3^rd^ most expressed proteins at 37°C and 42°C, respectively, but their ranks became 218^th^ and 82^nd^ when *L. monocytogenes* was cultivated at 10°C (Table S2). Another cold shock protein (GI: 16803404), on the contrary, was highly expressed at low temperature but the expression levels were dramatically decreased with the increase of growth temperature (Fig. S2). The expression level was also impacted by the growth stages. The expression levels of cold shock protein and GroES at the exponential phase were higher than those at the stationary phase. Meanwhile, GroEL and DnaK were more expressed at the stationary phase than the exponential phase (Fig. S2).

Several hypothetical proteins and enzymes involved in glycolysis were noticed in the highly expressed proteins. Glycolytic enzymes were expressed constantly in a growth-dependent manner. However, although the specific growth rates at 37°C and 42°C were the maximum, the expression level at 30°C was higher (Fig. S3A-S3D). Interestingly, three hypothetical proteins were determined as highly expressed proteins. The expression of the hypothetical protein lmo2223 encoded on GI: 16804262 was similar to the glycolytic enzymes and maintained a rank between 10 to 20 (Table S2). The other two hypothetical proteins were also continuously expressed but their ranks did not seem to have a correlation to the growth temperatures.

### The Summary of Protein Expressions

The list of proteins that quantitatively observed their expressions were summarized in the Supplementary Tables. Cell growth-related proteins that include ATP synthase complex, amino acid synthesis, cell growth and division, and lipid metabolism enzymes were summarized in Table S3. Table S4 contains the quantitative values of the protein expression involved in the heat shock and stress responses. The enzymes that constitute the central carbon catabolic pathways such as glycolysis, fermentation, pentose phosphate pathway, and amino sugar metabolism were listed in Table S5. Ribosomal proteins, tRNA synthesis, DNA/RNA polymerase, helicase, transcription & translation factors and other proteins involved in the protein synthesis were summarized in Table S6. Table S7 listed the protein for transport such as PTS and ABC transporters. The expression of fifteen different proteases was observed to include the aminopeptidase, clip protease family and endopeptidases (Table S8). Other proteins involved in the various functional activity of the cell were summarized in Table S9, and they included cellular mobility, redox balance, protein translocation, secondary metabolite synthesis, cellular signaling and regulation, nucleotide synthesis, cell wall synthesis, etc.

### Expression of Heat Shock and Stress Response Proteins

**Fig. 4:**
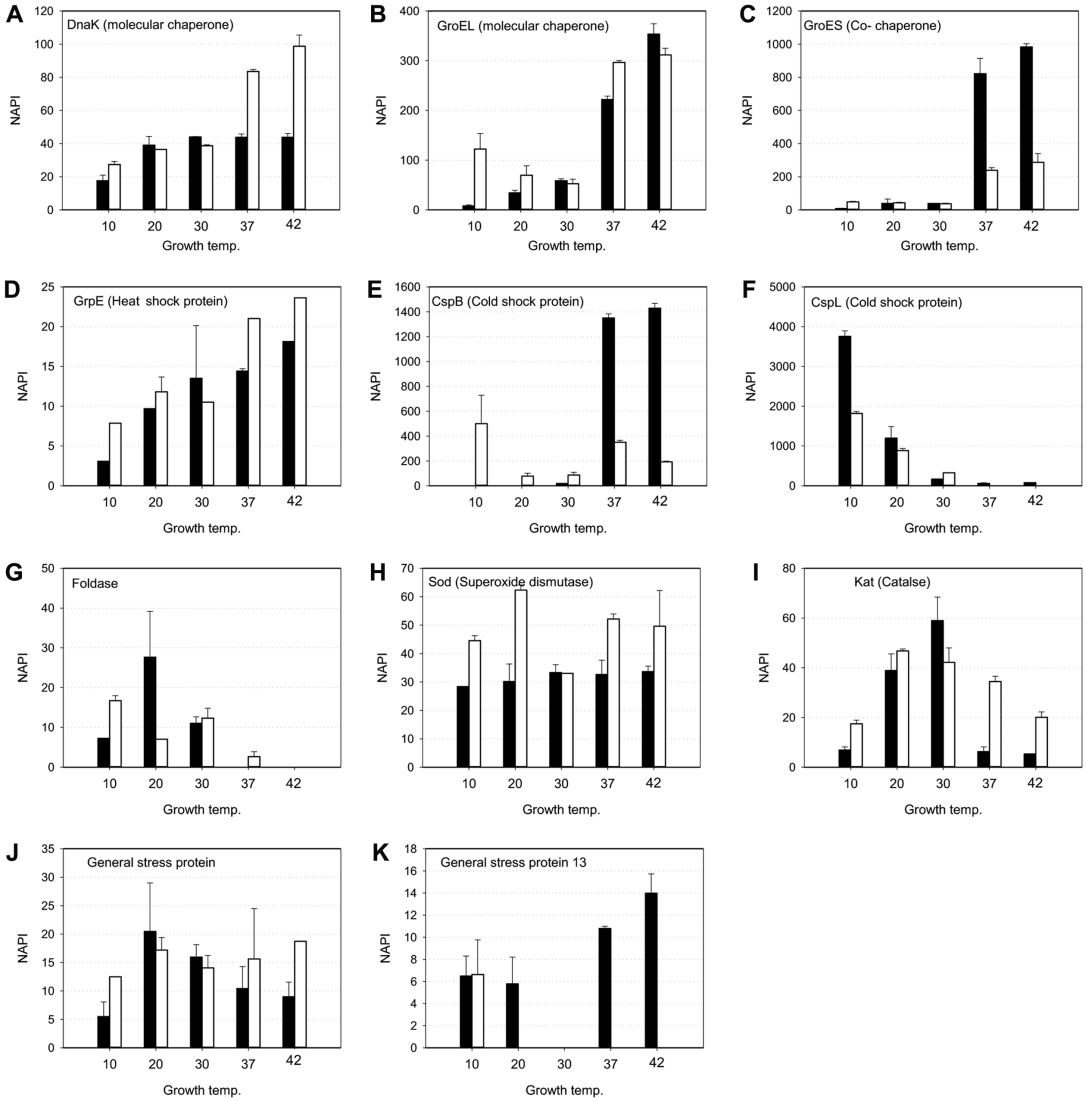
Expression of heat shock and stress response proteins in *L. monocytogenes*. (**A**) Molecular chaperone, DnaK (GI: 16803513), (**B**) molecular chaperone, GroEL (GI: 16804107), (**C**) co-chaperone, GroES (GI: 16804108), (**D**) heat shock protein, GrpE (GI: 16803514), (**E**) cold shock protein, CspB (GI: 16804055), (**F**) cold shock protein, CspL (GI: 16803404), (**G**) foldase (GI: 16804258), (**H**) superoxide dismutase, Sod (GI: 16803479), (**I**) catalase, Kat (GI: 16804822), (**J**) general stress protein (GI: 16803641), (**K**) general stress protein 13 (GI:16804407). Black and white boxes indicate the expression at exponential and stationary phase, respectively.

Among the crucial factors that enable *L. monocytogenes* to grow in the wide range of temperature were the stress response proteins including the series of heat and cold shock proteins [34-38]. The expressions of seven heat and cold shock proteins were determined by proteomic analysis showing strong temperature and growth stage dependency (Figs. 4A-4G). Chaperon proteins facilitate the refolding of damaged proteins under stress conditions [39, 40]. Both molecular chaperone DnaK and heat shock protein GrpE exhibited the increase of expression with increasing growth temperature (Figs. 4A and 4D). Interestingly, their expressions at the stationary phase were similar to or higher than in the exponential phase. Particularly, at low and high temperatures, expressions at the stationary phase were 1.5~2.5 fold higher than those at exponential phases. GroEL is a highly conserved protein that works with GroES to maintain protein integrity. [41, 42]. Two molecular chaperons, GroEL and GroES, were highly expressed when the growth temperature was high, above 37°C (Figs. 4B and 4C). The expressions of GroEL and GroES at 10°C were almost negligible at the exponential growth phase, however, the expressions at 42°C were increased 45-fold and 124-fold, respectively. The dramatic increase was observed only in the exponential phases. At stationary phase, though the expressions were increased with the increase of growth temperature as well, the fold ratios were between 1.5 to 2.5 only.

There were two cold shock proteins, CspB and CspL (CspA family), expressed during cell growth [43-45]. Expression of CspB (GI: 16804055) was highly induced during the growth in exponential phase at 37 and 42°C (Fig. 4E). At 10 and 20°C, no indication of the expressions was observed. Meanwhile, they became the second most expressed proteins in *L. monocytogenes* at 37 and 42°C. The expression of CspB exhibited different traits at the stationary phase. At low and high temperatures, the expression level increased showing 5~6-fold of that observed at 30°C. Another cold shock protein, CspL (GI:16803404), showed the opposite correlation to the other stress response proteins (Fig. 4F). It was highly expressed at low temperature and decreased its expression reaching negligible levels at 37 and 42°C. Although lower than those at the exponential phase, the expressions at the stationary phase showed similar traits.

Superoxide dismutase (Sod) and catalase (Kat) are the enzymes responsible for the oxidative stress of the cell [46-48]. The expression level of superoxide dismutase was maintained constant in general both at exponential and stationary phases (Figs. 4H and 4I). At stationary phases, the expression levels were 1.5~2.0-fold higher than those at exponential phases. Catalase is the enzyme that converts the hydrogen peroxide produced by SOD to water and oxygen molecules. The expression level was maximum at 30°C and decreased at low and high growth temperatures. When compared to exponential phases, expressions at the stationary phase were higher, particularly at high temperatures.

Foldase and two general stress response proteins were observed in their expressions (Figs. 4G, 4J, and 4K). Foldase expression was found at low temperatures and could not be observed at 37 and 42°C. The expressions of the general stress response proteins were observed at all temperatures but not definitive due to their low expression levels.

### Expression of Proteins for Cellular Mobility and Redox Balance

**Fig. 5:**
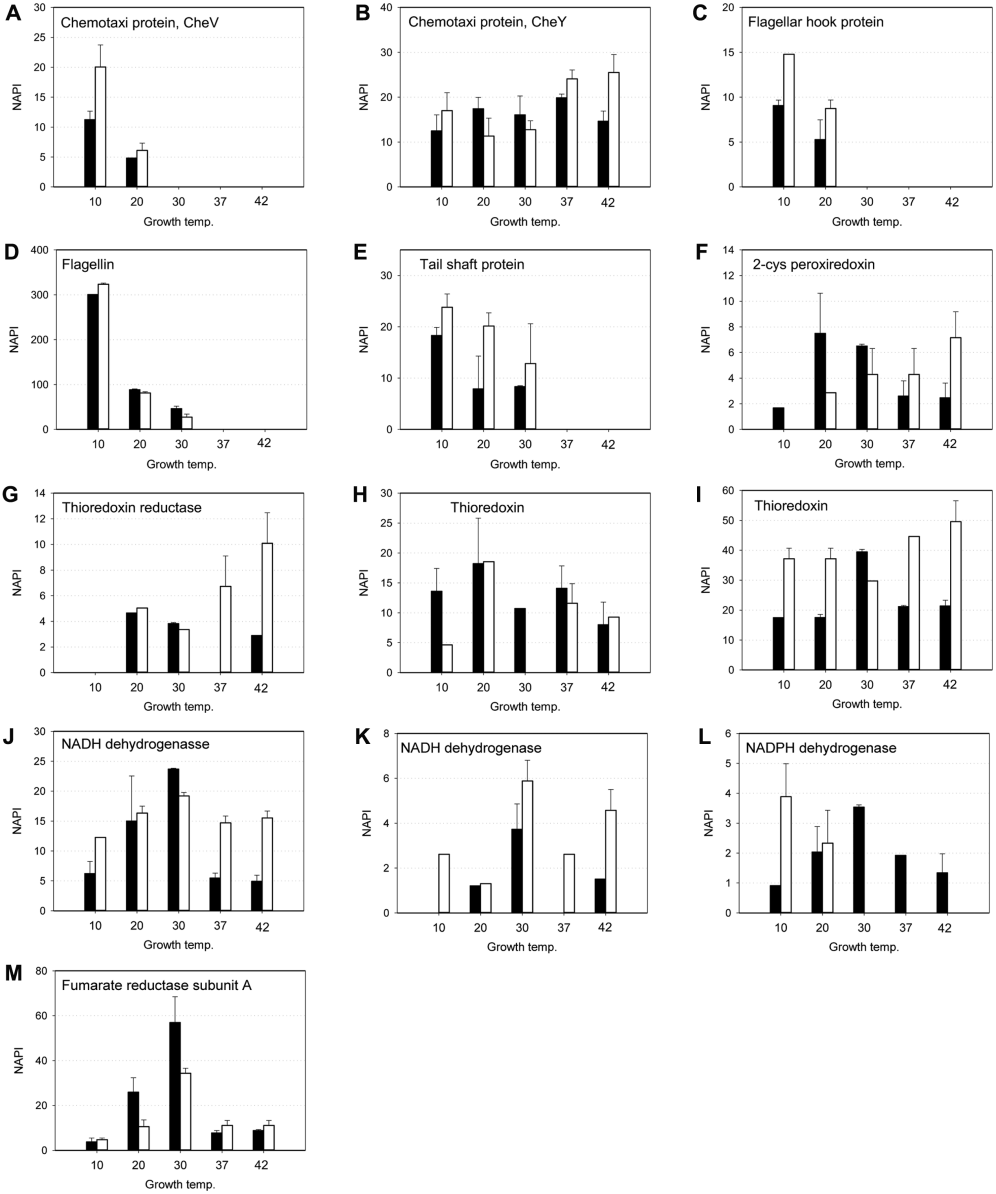
Expression of proteins related to the cellular mobility and redox balance of *L. monocytogenes*. Motility proteins: (**A**) chemotaxi protein, CheV (GI: 16803739), (**B**) chemotaxi protein, CheY (GI: 16804059), (**C**) flagella hook protein, FlaE (GI: 16802739), (**D**) flagellin, FlaA (GI: 16802732), (**E**) major tail shaft protein, (GI: 16804330). Redox balance protein: (**F**) 2-cys peroxiredoxin (GI: 16803644), (**G**) thioredoxin reductase, TrxB, (GI: 16804516), (**H**) thioredoxin, TrxA (GI: 16804191), (**I**) thioredoxin, TrxA (GI: 16803273), (**J**) NADH dehydrogenase (GI: 16804676), (**K**) NADH dehydrogenase (GI: 16804427), (**L**) NADPH dehydrogenase (GI: 16804509), (**M**) fumarate reductase subunit A (GI: 16802400) . Black and white boxes indicate the expression at exponential and stationary phase, respectively.

The expressions of several proteins were categorized by their functionalities (Fig. 5). CheV (GI: 16803739) and CheY (GI: 16804059) were the proteins involved in the chemical recognition activity of *L. monocytogenes* (Figs. 5A and 5B). The expression of CheV was observed at low temperature only, meanwhile, CheY was expressed constitutively regardless of growth temperatures or growth stages. The expressions of three flagella proteins were observed (Figs. 5C-5E). Flagellin proteins are the globular proteins that comprised the flagella tail that attached to the bacterial cell membrane through the flagellar hook and flagellar motor proteins [49, 50]. The expression of those three proteins was only induced at low temperatures. Expression was not observed above 30°C and the expression levels at 10°C of exponential and stationary phase were 6.5-fold and 11.8-fold higher, respectively than those observed at 30°C.

Fig. 5F to Fig. 5I described the protein expressions involved in the redox balance of the cell through thioredoxin and peroxiredoxin metabolism. The expression level of those proteins was relatively lower than other proteins, although expressions seemed to be constitutive. Membrane electron transfer chain (ETC) proteins were found to be expressed (Figs. 5J-5M). Expression pattern at the exponential phase was similar to the catalase and the glycolytic enzymes. The highest expression was observed at 30°C. With the increase of growth temperature from 10°C, expression of proteins was gradually increased, then the expression level was dropped significantly at 37 and 42°C. It suggested that the NADH reduction through ETC was coupled with the carbon dissimilation flux which seems to be linked to the catalase activity that alleviates the oxidative stresses.

### Expression of the Central Carbon Catabolic Pathway

**Fig. 6:**
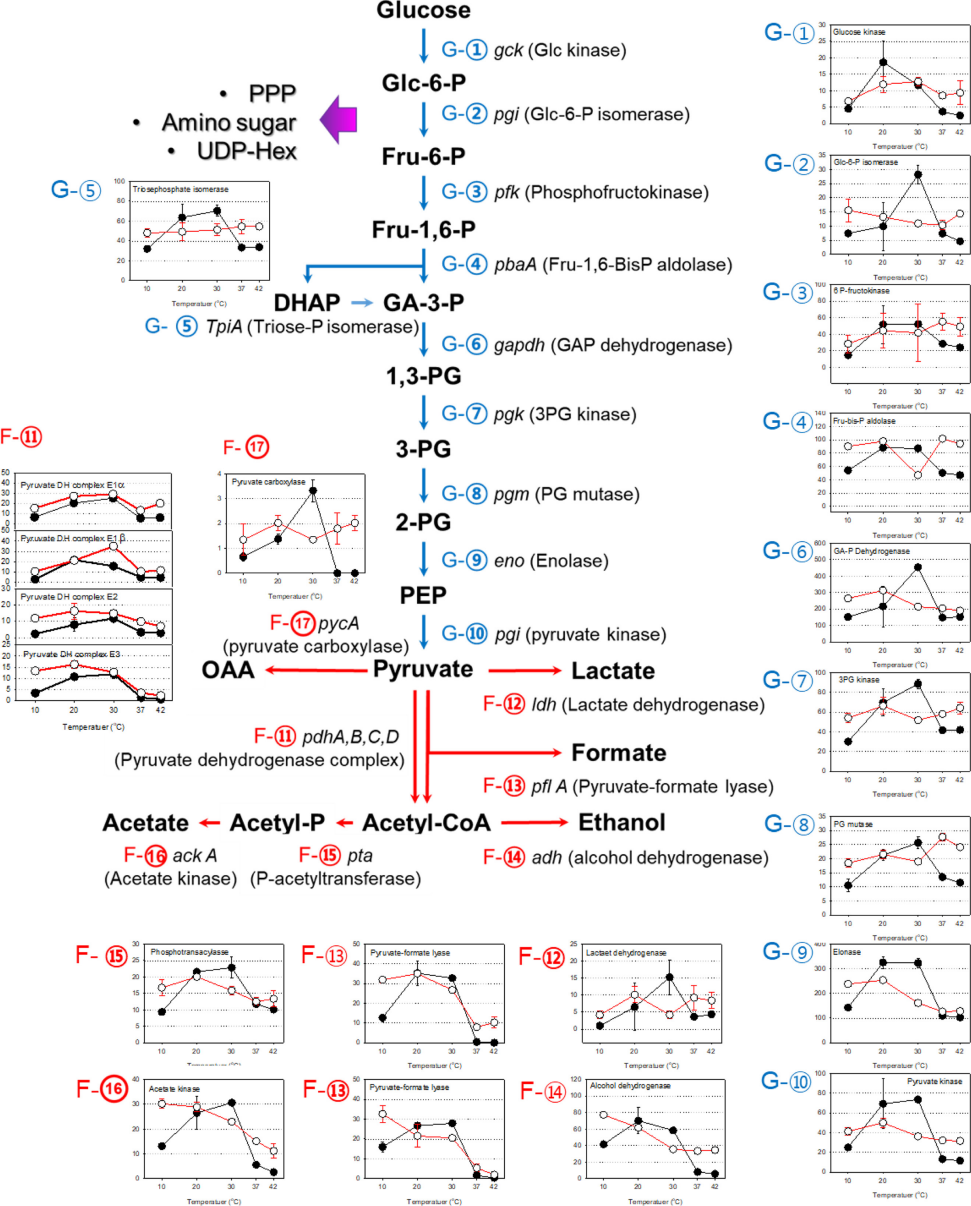
Suggested central carbon dissimilation pathway and their expressions in *L. monocytogenes*. Enzymes involved in the metabolic pathway was noted in the figures and their expression level was described in the graph which was linked by numbers. Black and white circle indicated the expression level at exponential and stationary phase, respectively.

The central carbon catabolic pathway was constructed from the protein expression data (Fig. 6 and Table S5). They included ten glycolytic enzymes that could catalyze glucose (C6) to two pyruvates (C3), four sets of enzymes that convert pyruvate (C3) to the various number of carbon metabolites from C4 to C1, and enzymes that process acetyl CoA (C2) metabolite to fermentation end products.

The overall expression patterns of enzymes at the exponential phase were similar in general. With increasing growth temperature, the expression levels were increased till 30°C. Some enzymes increased their expressions proportional to the increase of growth temperature, however, expression levels at 20°C were similar to those at 30°C mostly. The expression level at 30°C was 2~3 fold higher than that observed at 10°C. The amounts of expression at 37 and 42°C were similar and lower than or close to the expression level at 10°C.

The expression of catabolic enzymes at the stationary phase showed two different patterns. Mostly the expression was constitutive with similar NSAFs at the stationary phase. The range of the expression level at different temperatures was less than 2-fold. Fermentation and some glycolytic enzymes such as alcohol dehydrogenase, acetate kinase, pyruvate-formate lyase or enolase change their expression levels in a different fashion. The protein expression was highest at 10°C and gradually decreased with the increase of growth temperature.

A series of enzymes involved in glycolysis were identified in their expression in Fig. 6 (G①~G⑩). Among ten enzymes that catalyze glucose to pyruvate, glyceraldehyde-3-phosphate dehydrogenase and enolase were highly expressed, followed by 6-phosphofructokinase, fructose bisphosphate aldolase, 3-phosphoglycerate kinase, and pyruvate kinase.

There were four enzymes that convert pyruvate to different carbon metabolites. Pyruvate carboxylase could convert the three-carbon (C3) pyruvate to four-carbon (C4) oxaloacetate (OAA) by adding HCO_3_^-^. Though the observed expression level was very low, its response to the growth temperature was common to other glycolytic enzymes. Pyruvate could be oxidized to the same three-carbon (C3) end-product. Expression of lactic acid dehydrogenase (LDH) was observed in all temperatures, although the expression level seems lower than other fermentative enzymes. The expression of pyruvate dehydrogenase complex (PDC) that converts pyruvate (C3) to acetyl CoA (C2) and CO_2_ (C1) was found. At different growth temperatures, alpha and beta subunit of E1 (pyruvate dehydrogenase), E2 (dihydrolipoyl transacetylase) and E3 (dihydrolipoyl dehydrogenase) were expressed in a similar pattern. Pyruvate could be converted into two-carbon (C2) acetyl-CoA by pyruvate-formate lyase (PFL). Pyruvate-formate lyase breaks the pyruvate into acetyl-CoA and formate instead of CO_2_. Expressions of two independent PFLs were identified by proteomic analysis and their expression pattern over temperature was identical.

Acetyl-CoA could be fermented to acetate or ethanol. Expressions of phosphoacetyltransferase and acetate kinase were observed with a similar expression pattern over the temperatures and growth stages. One of the alcohol dehydrogenases was highly expressed constitutively. Considering the expression pattern in response to the temperature and growth stage, which was identical to pyruvate-formate lyase or acetate kinase, it might be the alcohol dehydrogenase that converts acetyl-CoA to ethanol.

It is noteworthy that *L. monocytogenes* is a facultative anaerobic bacterium and the cultivation was performed in aerobic conditions; however, enzymes involved in the TCA cycle were not clearly observed (Table S5). Aconitate hydratase was identified at a very low level and at only 20 to 30°C. Expression of fumarate reductase subunit A was observed but it seems that it was functioned as a part of the electron transfer chain system.

Enzymes for the pentose phosphate pathway were expressed, and 6-phosphogluconate dehydrogenase was expressed constitutively with an expression pattern that seemed similar to other glycolytic enzymes. Expressions of glucose-6-phosphate dehydrogenase, phosphopentomutase, and transketolase were identified as well. A series of key enzymes for the amino sugar metabolism were observed including glucosamine-fructose 6 phosphate aminotransferase, phosphoglucosamine mutase, and mannose 6-phosphate isomerase. Due to the low expression level, it could not be said definitively, however, that these enzymes showed dependency on the temperature or growth stages.

## Supplemental Materials



Supplementary data for this paper are available on-line only at http://jmb.or.kr.

## References

[1] Farber J, Peterkin P (1991). *Listeria monocytogenes*, a foodborne pathogen. Microbiol. Rev..

[2] Little C, Gillespie I (2008). Prepared salads and public health. J. Appl. Microbiol..

[3] Lungu B, Ricke S, Johnson M (2009). Growth, survival, proliferation and pathogenesis of *Listeria monocytogenes* under low oxygen or anaerobic conditions: a review. Anaerobe.

[4] Lund BM, O 'Brien SJ (2011). The occurrence and prevention of foodborne disease in vulnerable people. Foodborne Pathog. Dis..

[5] Schlech III WF 3rd (2000). Foodborne listeriosis. Clin. Infect. Dis..

[6] Low J, Donachie W (1997). A review of *Listeria monocytogenes* and listeriosis. Vet. J..

[7] Hamon M, Bierne H, Cossart P (2006). *Listeria monocytogenes*:a multifaceted model. Nat. Rev. Microbiol..

[8] te Giffel MC, Zwietering MH (1999). Validation of predictive models describing the growth of *Listeria monocytogenes*. Int. J. Food Microbiol..

[9] Pouillot R, Klontz KC, Chen Y, Burall LS, Macarisin D, Doyle M (2016). Infectious dose of *Listeria monocytogenes* in outbreak linked to ice cream, United States, 2015. Emerg. Infect. Dis..

[10] Kallipolitis BH, Ingmer H (2001). *Listeria monocytogenes* response regulators important for stress tolerance and pathogenesis. FEMS Microbiol. Lett..

[11] Walker S, Archer P, Banks JG (1990). Growth of *Listeria monocytogenes* at refrigeration temperatures. J. Appl. Bacteriol..

[12] Sorrells KM, Enigl DC, Hatfield JR (1989). Effect of pH, acidulant, time, and temperature on the growth and survival of *Listeria monocytogenes*. J. Food Prot..

[13] Control CfD, Prevention (2011). Multistate outbreak of listeriosis associated with Jensen Farms cantaloupe--United States, August-September 2011. MMWR. Morb. Mortal. Wkly Rep..

[14] Dewey-Mattia D, Manikonda K, Hall AJ, Wise ME, Crowe SJ (2018). Surveillance for foodborne disease outbreaks- United States, 2009-2015. MMWR Surveill. Summ..

[15] Jackson KA, Gould LH, Hunter JC, Kucerova Z, Jackson B (2018). Listeriosis outbreaks associated with soft cheeses, United States, 1998-2014. Emerg. Infect. Dis..

[16] Ding T, Iwahori Ji, Kasuga F, Wang J, Forghani F, Park M-S (2013). Risk assessment for *Listeria monocytogenes* on lettuce from farm to table in Korea. Food Control.

[17] Baek S-Y, Lim S-Y, Lee D-H, Min K-H, Kim C-M (2000). Incidence and characterization of *Listeria monocytogenes* from domestic and imported foods in Korea. J. Food Protect..

[18] Krüger E, Hecker M (1998). The first gene of the *Bacillus subtilis* clpC operon, ctsR, encodes a negative regulator of its own operon and other class III heat shock genes. J. Bacteriol..

[19] van der Veen S, Hain T, Wouters JA, Hossain H, de Vos WM, Abee T (2007). The heat-shock response of *Listeria monocytogenes* comprises genes involved in heat shock, cell division, cell wall synthesis, and the SOS response. Microbiology.

[20] Nelson KE, Fouts DE, Mongodin EF, Ravel J, DeBoy RT, Kolonay JF (2004). Whole genome comparisons of serotype 4b and 1/2a strains of the food-borne pathogen *Listeria monocytogenes* reveal new insights into the core genome components of this species. Nucleic Acids Res..

[21] B ayles DO, Annous B A, W ilkinson B J (1996). C old stress proteins induced in *Listeria monocytogenes* in response to temperature downshock and growth at low temperatures. Appl. Environ. Microbiol..

[22] Giotis ES, Muthaiyan A, Blair IS, Wilkinson BJ, McDowell DA (2008). Genomic and proteomic analysis of the Alkali Tolerance Response (AlTR) in *Listeria monocytogenes* 10403S. BMC Microbiol..

[23] Zhu W, Smith JW, Huang C-M (2009). Mass spectrometrybased label-free quantitative proteomics. J. Biomed. Biotechnol..

[24] Agoston R, Soni K, Jesudhasan PR, Russell WK, MohácsiFarkas C, Pillai SD (2009). Differential expression of proteins in *Listeria monocytogenes* under thermotolerance-inducing, heat shock, and prolonged heat shock conditions. Foodborne Pathog. Dis..

[25] Cacace G, Mazzeo MF, Sorrentino A, Spada V, Malorni A, Siciliano RA (2010). Proteomics for the elucidation of cold adaptation mechanisms in *Listeria monocytogenes*. J. Proteomics.

[26] Pittman JR, Buntyn JO, Posadas G, Nanduri B, Pendarvis K, Donaldson JR (2014). Proteomic analysis of cross protection provided between cold and osmotic stress in *Listeria monocytogenes*. J. Proteome Res..

[27] Washburn MP, Ulaszek R, Deciu C, Schieltz DM, Yates JR, 3rd (2002). Analysis of quantitative proteomic data generated via multi dimensional protein identification technology. Anal. Chem..

[28] Christensen DP, Benson AK, Hutkins RW (1999). Mutational analysis of the role of HPr in *Listeria monocytogenes*. Appl. Environ. Microbiol..

[29] Burke TP, Portnoy DA (2016). SpoVG is a conserved RNAbinding protein that regulates *Listeria monocytogenes* lysozyme resistance, virulence, and swarming motility. mBio..

[30] Kunze G, Zipfel C, Robatzek S, Niehaus K, Boller T, Felix G (2004). The N terminus of bacterial elongation factor Tu elicits innate immunity in Arabidopsis plants. Plant Cell..

[31] Agrawal RK, Sharma MR, Kiel MC, Hirokawa G, Booth TM, Spahn CM (2004). Visualization of ribosome-recycling factor on the *Escherichia coli* 70S ribosome: functional implications. Proc. Natl. Acad. Sci..

[32] Rouquette C, Ripio MT, Pellegrini E, Bolla JM, Tascon RI, Vázquez-Boland JA (1996). Identification of a ClpC ATPase required for stress tolerance and in vivo survival of *Listeria monocytogenes*. Mol. Microbiol..

[33] Cotter PD, Emerson N, Gahan CG, Hill C (1999). Identification and disruption of lisRK, a genetic locus encoding a twocomponent signal transduction system involved in stress tolerance and virulence in *Listeria monocytogenes*. J. Bacteriol..

[34] Hanawa T, Fukuda M, Kawakamis H, Hirano H, Kamiya S, Yamamoto T (1999). The *Listeria monocytogenes* DnaK chaperone is required for stress tolerance and efficient phagocytosis with macrophages. Cell Stress Chaperones.

[35] Hébraud M, Guzzo J (2000). The main cold shock protein of *Listeria monocytogenes* belongs to the family of ferritin-like proteins. FEMS Microbiol. Lett..

[36] Thieringer HA, Jones PG, Inouye M (1998). Cold shock and adaptation. Bioessays.

[37] Jones PG, Inouye M (1994). The cold-shock response-a hot topic. Mol. Microbiol..

[38] Sharp FR, Massa SM, Swanson RA (1999). Heat-shock protein protection. Trends Neurosci..

[39] Schröder H, Langer T, Hartl F, Bukau B (1993). DnaK, DnaJ and GrpE form a cellular chaperone machinery capable of repairing heat-induced protein damage. EMBO J..

[40] Georgopoulos C, Liberek K, Zylicz M, Ang D (1994). 9 Properties of the heat shock proteins of *Escherichia coli* and the autoregulation of the heat shock response. Cold Spring Harbor Monograph Archive..

[41] Segal G, Ron EZ (1996). Regulation and organization of the groE and dnaK operons in Eubacteria. FEMS Microbiol. Lett..

[42] Hendrick JP, Hartl F-U (1993). Molecular chaperone functions of heat-shock proteins. Annu. Rev. Biochem..

[43] Schmid B, Klumpp J, Raimann E, Loessner MJ, Stephan R, Tasara T (2009). Role of cold shock proteins in growth of *Listeria monocytogenes* under cold and osmotic stress conditions. Appl. Environ. Microbiol..

[44] Yamanaka K, Fang L, Inouye M (1998). The CspA family in *Escherichia coli*: multiple gene duplication for stress adaptation. Mol. Microbiol..

[45] Phadtare S, Alsina J, Inouye M (1999). Cold-shock response and cold-shock proteins. Curr. Opin. Microbiol..

[46] Day WA, Sajecki JL, Pitts TM, Joens LA (2000). Role of catalase in *Campylobacter jejuni* intracellular survival. Infect. Immun..

[47] Lynch M, Kuramitsu H (2000). Expression and role of superoxide dismutases (SOD) in pathogenic bacteria. Microb. Infect..

[48] Fisher CW, Lee D, Dodge B-A, Hamman KM, Robbins JB, Martin SE (2000). Influence of catalase and superoxide dismutase on ozone inactivation of *Listeria monocytogenes*. Appl. Environ. Microbiol..

[49] Laurent V, Loisel TP, Harbeck B, Wehman A, Gröbe L, Jockusch BM (1999). Role of proteins of the Ena/VASP family in actin-based motility of *Listeria monocytogenes*. J. Cell Biol..

[50] Kathariou S, Mizumoto C, Kanenaka R, Allen R, Fok A (1995). Repression of motility and flagellin production at 37°C is stronger in *Listeria monocytogenes* than in the nonpathogenic species Listeria innocua. Can. J. Microbiol..

