# Supplementary material for: Comparative Whole Cell Proteomics of Listeria monocytogenes at Different Growth Temperatures
Source: J Microbiol Biotechnol. 2019 Dec 2;30(2):259–70. doi: 10.4014/jmb.1911.11027 (PMC9728365; doi:10.4014/jmb.1911.11027)
Supplement: Supplementary file 1 [file JMB-30-2-259-supple.pdf]

J. Microbiol. Biotechnol.
https://doi.org/10.4014/jmb.1911.11027

jmb

Supplementary Figure 1 The expression of highly expressed proteins. Group 1: (A) DNA binding protein-HU, (B) phosphor carrier protein HPr (GI: 16803042), (C) RRF (Ribosomal recycling protein, GI: 16803354) (D) Ferritin (non-heme iron-binding, GI: 16802983), (E) EF-Tu (Elongation factor-Tu, GI: 16804690), (F) ET-Ts (Elongation factor-Ts, GI: 16803697), (G) Regulatory protein (SpoVS family: GI 16802242), (H) Regulatory protein (SpoVS family: GI 16802243). Block and white bar indicates the exponential and stationary phase samples, respectively

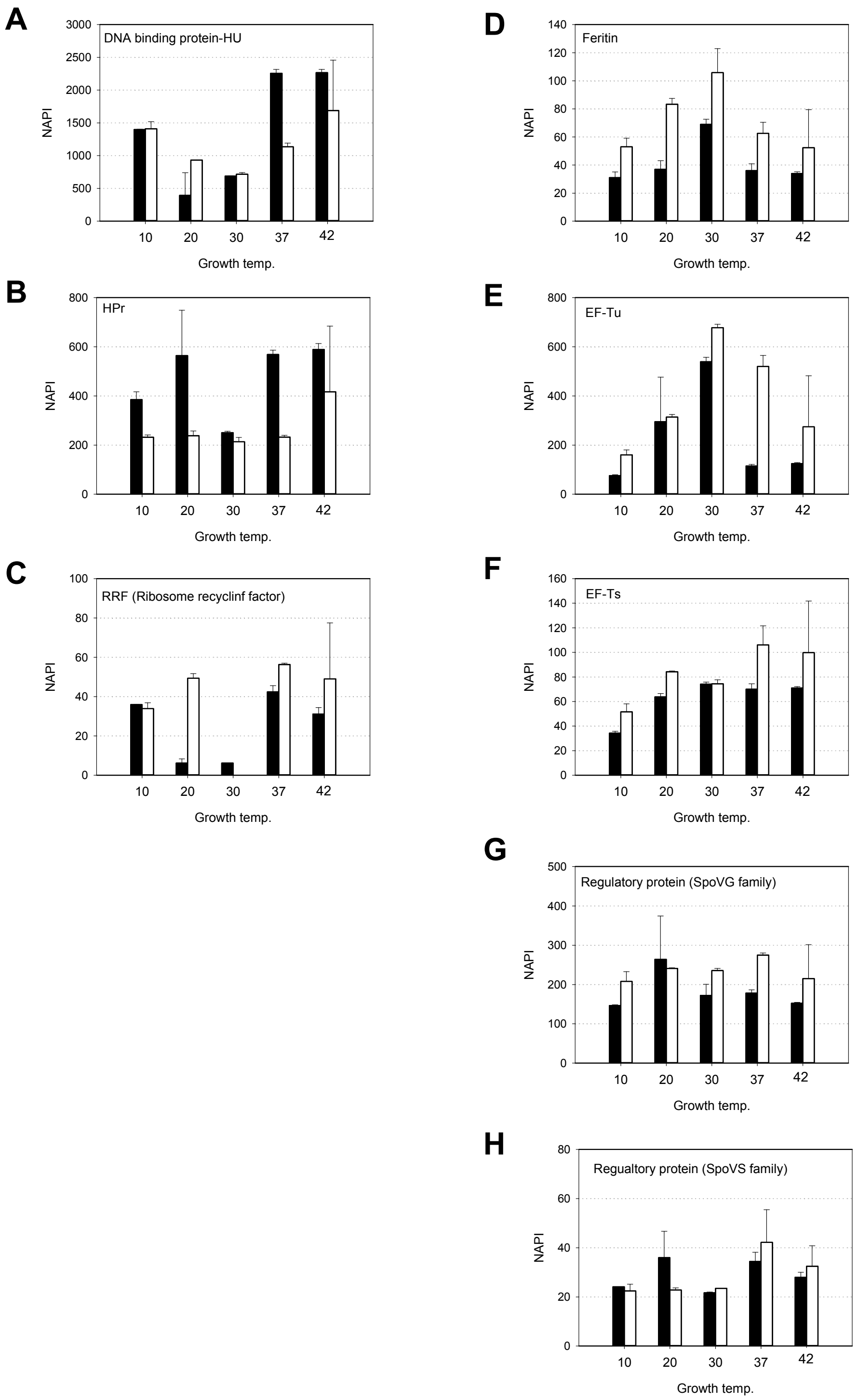

Supplementary Figure 2 The expression of highly expressed proteins. Group 2: (A) Cold shock protein (GI 16804055), (B) GroES (GI: 16804108), (C) GroEL (GI: 16804107), (D) DnaK (GI: 16803513), (E) Cold shock protein (GI: 16803404). Block and white bar indicates the exponential and stationary phase samples, respectively

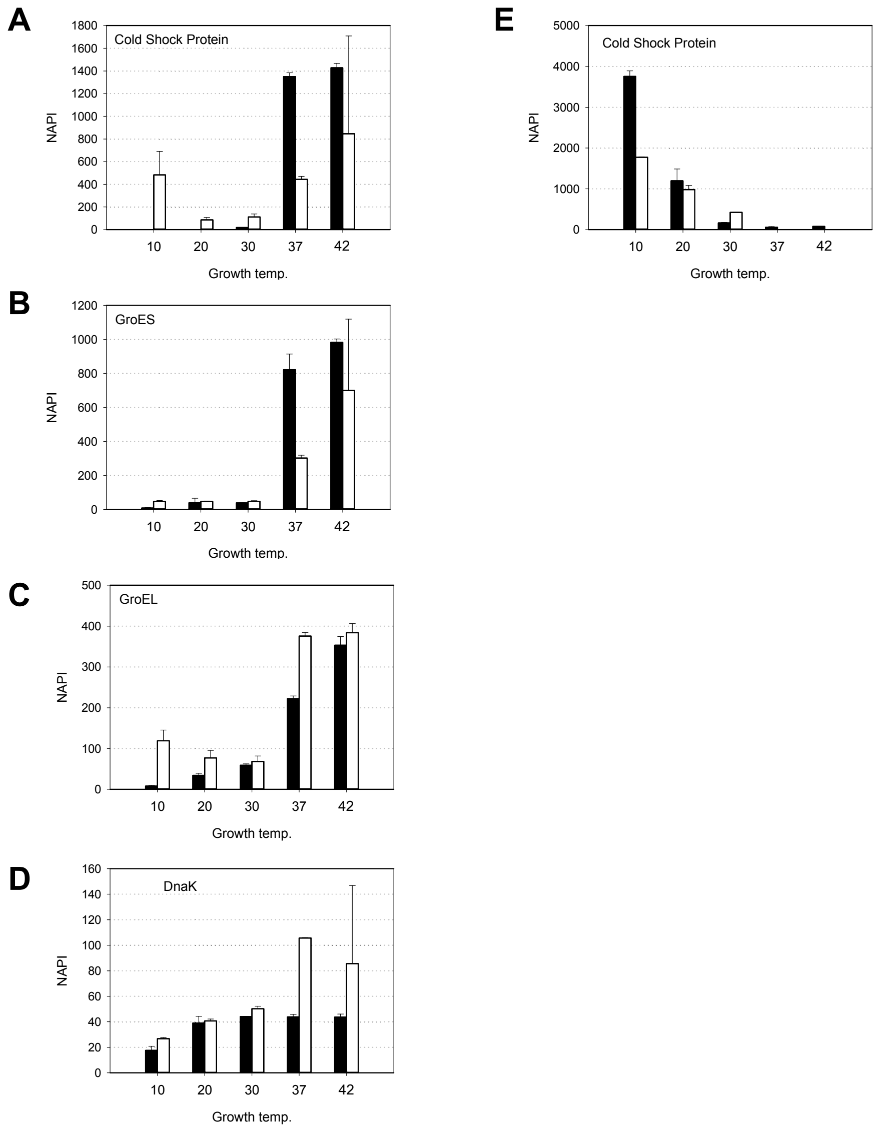

Supplementary Figure 3 The expression of highly expressed proteins. Group 3: (A) Fructose-6-phosphate aldolase (GI: 16804594), (B) Glyceraldehyde dehydrogenase (GI: 16804497), (C) Phosphoglycerate kinase (GI: 16804496), (D) Enolase (GI: 16804493), (E) Hypothetical protein lmo2223 (GI: 16804262), (F) hypothetical protein lmo1008 (GI: 16803068), (G) hypothetical protein lmo1008 (GI:10683048). Block and white bar indicates the exponential and stationary phase samples, respectively

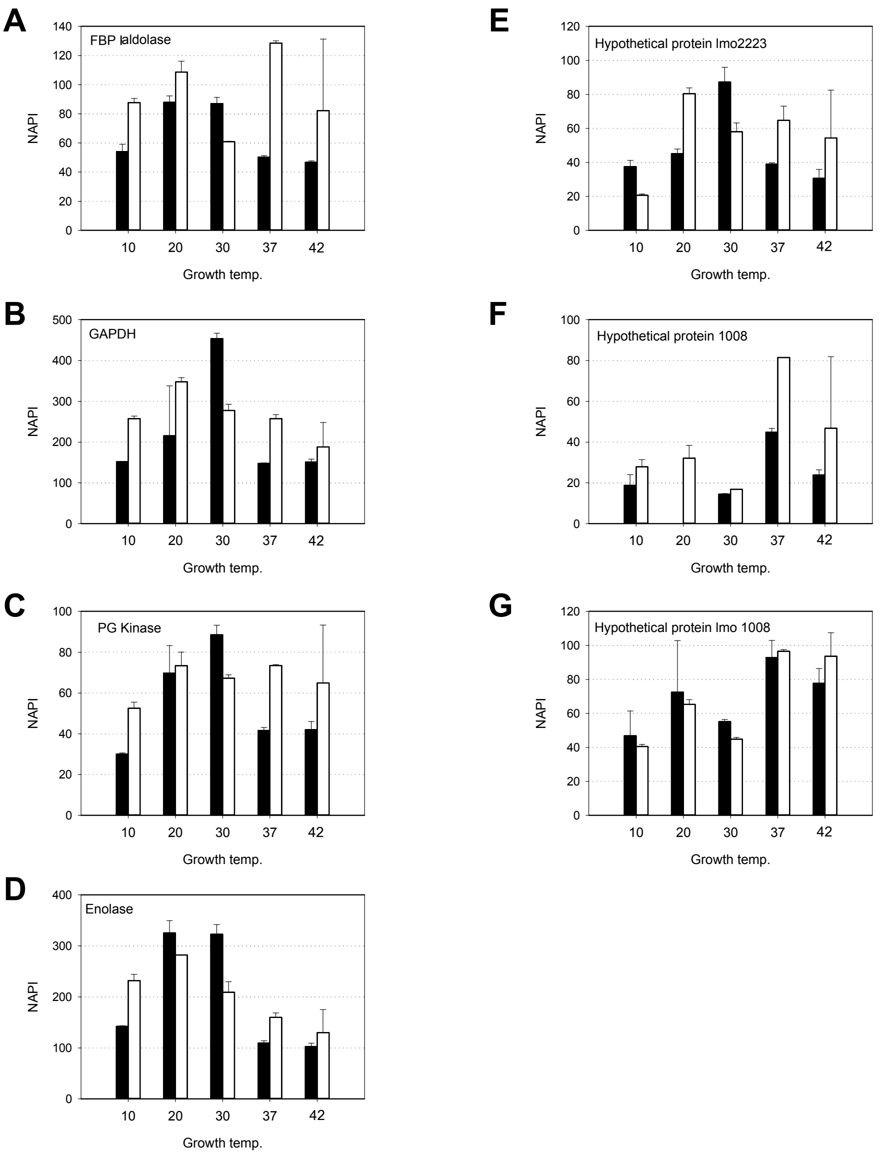

Supplementary Table 1. The summary of proteomics analysis results

Temperature	Growth phase	Total spectra assigned		Total unique assigned		Number of identified proteins	
		1[a]	2	1	2	1	2
10 ℃	Exponential	5220	5157	1107	1085	367	357
	Stationary	3906	4342	1149	1143	371	377
20 ℃	Exponential	4931	2688	1219	789	411	305
	Stationary	4170	3922	1172	1111	380	375
30 ℃	Exponential	4673	2930	1291	976	419	360
	Stationary	3670	3662	1140	1132	385	377
37 ℃	Exponential	5143	5236	1009	993	346	333
	Stationary	3932	3843	1146	1119	370	367
42 ℃	Exponential	5625	5561	954	943	333	317
	Stationary	3906	3888	1149	1112	371	366

[a] Experiments were performed in duplicate. The number means repeat time.

Supplementary Table 2. The expression level of top 20 most expressed proteins at 37℃ exponential phase.

Rank[a]	GI[b]	Name		NSAF X 10000 (Ave ± SD) [c]									
		Gene	Protein	Exponential phase					Stationary phase				
				10℃	20℃	30℃	37℃	42℃ E	10℃	20℃	30℃	37℃	42℃
1	16803973	*hup*	DNA-binding protein HU	1399 ± 4	392 ± 346	687 ± 4	2259 ± 57	2267 ± 50	1410 ± 109	932 ± 3	716 ± 27	1136 ± 54	1687 ± 770
2	16804055	*cspB*	cold-shock protein	0 ± 0	0 ± 0	18 ± 0	1350 ± 33	1429 ± 40	484 ± 207	87 ± 21	112 ± 26	444 ± 26	846 ± 864
3	16804108	*groES*	co-chaperonin GroES	8 ± 2	40 ± 26	39 ± 1	822 ± 92	983 ± 19	47 ± 5	47 ± 3	48 ± 4	303 ± 17	700 ± 419
4	16803042	*ptsH*	phosphocarrier protein HPr	385 ± 32	565 ± 185	251 ± 5	569 ± 18	589 ± 24	232 ± 9	238 ± 19	214 ± 18	232 ± 8	417 ± 267
5	16804107	*groEL*	molecular chaperone GroEL	8 ± 2	34 ± 5	59 ± 4	222 ± 7	353 ± 21	119 ± 27	77 ± 18	69 ± 14	375 ± 9	384 ± 22
6	16802243		regulatory protein SpoVG	147 ± 2	264 ± 110	172 ± 28	179 ± 8	152 ± 3	208 ± 25	241 ± 2	236 ± 6	275 ± 6	215 ± 86
7	16804497	*gapdh*	glyceraldehyde-3-phosphate dehydrogenase	152 ± 1	216 ± 123	454 ± 13	148 ± 1	151 ± 7	258 ± 6	348 ± 10	278 ± 15	258 ± 10	188 ± 60
8	16804690	*tuf*	elongation factor Tu	76 ± 4	295 ± 182	539 ± 18	116 ± 6	124 ± 5	161 ± 20	314 ± 11	678 ± 14	520 ± 45	275 ± 208
9	16804493	*eno*	phosphopyruvate hydratase	142 ± 1	326 ± 24	323 ± 19	110 ± 4	103 ± 7	232 ± 13	282 ± 0	209 ± 21	160 ± 9	130 ± 45
10	16803068		hypothetical protein lmo1008	47 ± 15	73 ± 30	55 ± 1	93 ± 10	78 ± 9	41 ± 1	65 ± 3	45 ± 1	97 ± 1	94 ± 14
11	16803697	*tsf*	elongation factor Ts	34 ± 2	64 ± 3	74 ± 2	70 ± 4	71 ± 1	52 ± 7	84 ± 1	74 ± 3	106 ± 16	100 ± 42
12	16803404	*csp*	cold-shock protein	3755 ± 139	1199 ± 289	166 ± 10	58 ± 19	79 ± 3	1772 ± 14	982 ± 102	424 ± 3	0 ± 0	0 ± 0
13	16804594	*fbaA*	fructose-1,6-bisphosphate aldolase	54 ± 5	88 ± 4	87 ± 4	50 ± 1	47 ± 1	88 ± 3	109 ± 7	61 ± 0	129 ± 2	82 ± 49
14	16803048		hypothetical protein lmo1008	19 ± 5	0 ± 0	15 ± 0	45 ± 2	24 ± 3	28 ± 4	32 ± 6	17 ± 0	82 ± 0	47 ± 35
15	16803513	*dnaK*	molecular chaperone DnaK	18 ± 3	39 ± 5	44 ± 0	44 ± 2	44 ± 2	27 ± 1	41 ± 2	50 ± 2	106 ± 0	86 ± 61
16	16803354		ribosome recycling factor	36 ± 0	6 ± 2	6 ± 0	42 ± 3	31 ± 3	34 ± 3	49 ± 2	0 ± 0	56 ± 1	49 ± 29
17	16804496	*pgk*	phosphoglycerate kinase	30 ± 1	70 ± 14	89 ± 5	42 ± 2	42 ± 4	53 ± 3	73 ± 7	67 ± 2	74 ± 0	65 ± 28
18	16804262		hypothetical observed protein lmo2223	38 ± 4	45 ± 3	87 ± 9	39 ± 1	31 ± 5	21 ± 1	80 ± 3	58 ± 5	65 ± 8	54 ± 28
19	16802983	*fri*	non-heme iron-binding ferritin	31 ± 4	37 ± 6	69 ± 4	36 ± 5	34 ± 1	53 ± 6	83 ± 4	106 ± 17	63 ± 8	52 ± 27
20	16802242		regulatory protein SpoVG	24 ± 0	36 ± 11	22 ± 0	34 ± 4	28 ± 2	22 ± 3	23 ± 1	24 ± 0	42 ± 13	33 ± 8

Rank[a] was calculated based on the expression at the exponential phase of 37°C cultivation.

Gi[b]: gene accession number (pubmed)

Supplementary Table 3. The summary of cell growth-related protein expressions

GI	Gene	Product	Exponential					Stationary				
			10℃	20℃	30℃	37℃	42℃	10℃	20℃	30℃	37℃	42℃
**ATP synthase**												
16804569	*atpA*	ATP synthase F0F1 subunit epsilon	9.8 ± 0.0	25.5 ± 11.5	26.7 ± 0.3	10.2 ± 0.2	9.0 ± 0.0	27.1 ± 4.4	26.6 ± 6.6	20.4 ± 0.7	25.6 ± 2.2	20.9 ± 3.0
16804566	*atpC*	ATP synthase F0F1 subunit beta	4.6 ± 0.0	0.0	4.4 ± 0.1	4.8 ± 0.1	2.2	7.8	3.9		7.8	3.9
16804567	*atpD*	ATP synthase F0F1 subunit alpha	18.7 ± 3.3	44.7 ± 3.6	53.2 ± 4.2	13.9 ± 0.8	13.8 ± 1.3	46.8 ± 6.3	45.7 ± 0.0	45.7 ± 3.2	38.4 ± 2.4	35.7 ± 4.7
16804571	*atpF*	ATP synthase F0F1 subunit delta	1.7	0.0	10.1 ± 0.2	0.0	0.0	4.4 ± 2.1	5.9 ± 0.0		3.0	3.0
16804570	*atpH*	ATP synthase F0F1 subunit B	10.8 ± 1.1	10.4	8.1 ± 2.5	10.4 ± 0.2	6.5 ± 2.3	17.0 ± 0.0	11.3 ± 4.0	5.7 ± 0.0	8.5 ± 4.0	11.3 ± 4.0
**Aminos acid synthesis**												
16803018		branched-chain amino acid aminotransferase	7.7 ± 3.2	10.7 ± 7.1	13.1 ± 1.0	7.0 ± 0.8	5.3 ± 1.2	12.3 ± 2.2	13.1 ± 1.1	10.8 ± 2.2	22.3 ± 1.1	28.4 ± 3.3
16802269	*cysK*	cysteine synthase	11.0 ± 0.8	16.6 ± 2.1	18.3 ± 0.4	20.7 ± 1.1	19.5 ± 1.5	20.5 ± 1.3	22.3 ± 1.3	18.8 ± 3.8	30.4 ± 5.1	33.9 ± 0.0
16802896	*ddl*	D-alanyl-alanine synthetase A	0.0	5.0 ± 0.3	1.6	0.0	0.0		4.2 ± 2.0	2.8	5.0 ± 1.0	7.8 ± 1.0
16803475	*dapA*	dihydrodipicolinate synthase	0.0	24.8 ± 8.8	31.2 ± 2.3	19.0 ± 2.8	18.4 ± 3.7	11.0 ± 0.0	25.6 ± 15.5	11.0 ± 5.2	24.7 ± 3.9	24.7 ± 1.3
16803659		D-amino acid aminotransferase	3.1	6.0	4.0	2.2	1.0	7.1	8.9 ± 2.5	1.8 ± 0.0	3.5	4.4 ± 1.3
16803704	*metK*	metadenosylmethionine synthetase	0.8 ± 0.0	1.7 ± 0.7	2.2 ± 1.0	0.8 ± 0.0	0.0	1.3	1.3 ± 0.0	2.0 ± 0.9	3.3 ± 0.9	2.0 ± 0.9
**Cell Growth**												
16804072	*ftsA*	cell division protein FtsA	0.7	1.2	5.7 ± 1.9	0.0	0.0	1.3	3.8 ± 1.8	6.3	6.3 ± 1.8	4.4 ± 0.9
16803811	*ftsA*	cell division protein FtsA	3.6	0.0	0.0	0.0	0.0					
16804071	*ftsZ*	cell division protein FtsZ	18.4 ± 1.7	27.0 ± 1.9	23.0 ± 6.1	18.7 ± 2.8	14.0 ± 2.8	37.6 ± 0.0	37.6 ± 3.9	22.3 ± 5.9	29.9 ± 6.9	31.3 ± 3.0
16804563	*mbl*	rod shape-determining protein MreB	1.0	15.9 ± 7.0	19.3 ± 4.3	2.5 ± 0.7	2.3 ± 0.7	7.3 ± 1.1	16.2 ± 2.3	23.4 ± 3.4	14.5 ± 0.0	11.3 ± 0.0
16803588	*mreB*	rod shape-determining protein MreB	18.1 ± 2.6	33.5 ± 20.2	41.4 ± 3.0	10.9 ± 1.2	10.2 ± 0.0	32.4 ± 2.3	34.8 ± 3.4	38.9 ± 0.0	21.9 ± 3.4	17.0 ± 1.1
16802266	*ftsH*	cell division protein FtsH	1.3 ± 0.0	3.7 ± 0.3	5.6 ± 0.5	0.5	0.2	1.9 ± 0.5	3.0 ± 1.1	5.7 ± 0.5	3.4 ± 0.5	2.6 ± 0.5
16803843	*ftsY*	cell division protein FtsY [	0.0	0.0	5.5	0.0	0.0			1.6	1.6	
16804827	*parB*	partition protein ParB homolg	0.0	0.0	0	1.6 ± 0.7	1.5 ± 0.7		2.7 ± 1.3	3.6	5.4 ± 0.0	4.5 ± 1.3
**Lipid metabolism**												
16803847	*fabG*	3-ketoacyl-ACP reductase	12.3 ± 2.8	34.1 ± 6.1	30.0 ± 4.2	14.7 ± 1.6	10.7 ± 4.4	15.4 ± 0.0	16.5 ± 1.6	30.7 ± 3.1	14.3 ± 4.7	13.2 ± 3.1
16804240		3-oxoacyl-ACP synthase	2.7 ± 0.5	11.5 ± 0.7	11.1 ± 0.8	3.9 ± 1.0	3.0 ± 1.0	3.9 ± 0.0	5.8 ± 2.8	12.3 ± 2.8	6.5 ± 0.0	5.2 ± 3.7
16802828		ACP phosphodiesterase	7.3 ± 0.0	22.2 ± 7.5	11.3 ± 0.2	3.8 ± 1.0	3.6 ± 1.0	5.0	18.7 ± 5.3	10.0 ± 3.5	5.0	7.5 ± 0.0
16803848	*fabD*	ACP S-malonyltransferase	3.6 ± 2.2	3.9 ± 1.3	5.0 ± 1.3	3.7 ± 0.7	2.0	6.1 ± 1.2	4.4 ± 3.7	3.5	3.5 ± 0.0	7.9 ± 6.2
16803010		enoyl-ACP reductase	1.2	17.8 ± 4.0	18.5 ± 2.9	2.5 ± 0.0	2.3 ± 0.0	5.1 ± 1.4	8.1 ± 2.9	12.2 ± 0.0	8.1 ± 0.0	7.1 ± 1.4
16802399		fatty-acid--CoA ligase	0.0	1.8 ± 0.1	5.1 ± 0.7	0.0	0.0	2.0	2.5 ± 0.7	2.5 ± 0.7		2.0 ± 1.4

Supplementary Table 4. The summary of heat shock and stress response protein expressions

GI	Gene	Product	Exponential					Stationary				
			10℃	20℃	30℃	37℃	42℃	10℃	20℃	30℃	37℃	42℃
16803513	*dnaK*	molecular chaperone DnaK	17.6 ± 3.3	39.0 ± 5.3	44.0 ± 0.3	43.8 ± 2.0	43.7 ± 2.3	27.4 ± 1.8	36.5 ± 0.0	38.7 ± 0.6	83.5 ± 1.2	98.7 ± 6.8
16804107	*groEL*	molecular chaperone GroEL	8.0 ± 2.1	34.3 ± 5.2	58.8 ± 3.7	222.1 ± 6.9	353.3 ± 21.0	122.4 ± 31.2	69.7 ± 19.2	52.7 ± 9.2	296.5 ± 3.5	311.6 ± 13.5
16804108	*groES*	co-chaperonin GroES	8.4 ± 2.4	39.5 ± 26.1	38.9 ± 0.8	822.1 ± 91.8	983.4 ± 19.1	48.4 ± 4.0	42.7 ± 4.0	37.0 ± 4.0	239.1 ± 16.1	287.5 ± 52.3
16804055	*cspB*	cold-shock protein	0.0	0.0	18.2	1350.4 ± 33.4	1428.6 ± 39.5	500.2 ± 228.4	78.8 ± 22.3	86.6 ± 22.3	350.5 ± 16.7	193.0 ± 5.6
16803404	*cspL*	cold-shock protein	3755.0 ± 139.2	1198.7 ± 288.5	165.8 ± 9.9	57.5 ± 19.3	79.2 ± 3.4	1819.6 ± 44.6	882.2 ± 55.7	326.9 ± 5.6		
16802268	*hslO*	heat shock protein 33	0.0	1.7	0.0	0.0	0.0					3.6
16803514	*grpE*	heat shock protein GrpE	3.1	9.7	13.5 ± 6.6	14.4 ± 0.3	18.1 ± 0.1	7.9 ± 0.0	11.8 ± 1.9	10.5	21.0 ± 0.0	23.6 ± 0.0
16804258		foldase	7.2 ± 0.0	27.6 ± 11.6	11.0 ± 1.7	0.0	0.0	16.7 ± 1.2	7.0 ± 0.0	12.3 ± 2.5	2.6 ± 1.2	
16804822	*kat*	catalase	6.9 ± 1.3	38.9 ± 6.7	59.0 ± 9.5	6.3 ± 1.9	5.3 ± 0.0	17.5 ± 1.5	46.8 ± 0.7	42.2 ± 5.8	34.5 ± 2.2	20.1 ± 2.2
16803479	*sod*	superoxide dismutase	28.4 ± 0.1	30.2 ± 6.2	33.3 ± 2.8	32.6 ± 5.0	33.6 ± 2.0	44.5 ± 1.8	62.3 ± 1.8	33.1 ± 0.0	52.2 ± 1.8	49.6 ± 12.6
16803641		general stress protein	5.5 ± 2.6	20.5 ± 8.6	16.0 ± 2.2	10.4 ± 3.8	9.0 ± 2.6	12.5 ± 0.0	17.2 ± 2.2	14.1 ± 2.2	15.6 ± 8.8	18.8 ± 0.0
16804407		general stress protein 13	6.5 ± 1.8	5.8 ± 2.4	0.0	10.8 ± 0.2	14.0 ± 1.8	6.6 ± 3.1				

Supplementary Table 5. The summary of the central carbon catabolic enzymes expressions

GI	Gene	Product	Exponential					Stationary				
			10℃	20℃	30℃	37℃	42℃	10℃	20℃	30℃	37℃	42℃
**Glycolysis**												
16803379	gck	glucose kinase	4.5 ± 0.7	18.7 ± 6.4	11.6 ± 0.3	3.6 ± 0.7	2.5 ± 0.7	6.8 ± 0.0	11.9 ± 2.4	12.8 ± 1.2	8.5 ± 0.0	9.4 ± 3.6
16804405	pgi	glucose-6-phosphate isomerase	7.4 ± 0.9	9.9 ± 8.5	28.2 ± 3.4	7.4 ± 0.6	4.6 ± 0.9	15.6 ± 4.1	13.3 ± 0.8	10.9 ± 0.8	10.4 ± 1.6	14.4 ± 0.8
16803611	pfkA	6-phosphofructokinase	14.7 ± 0.1	51.7 ± 23.1	52.3 ± 0.2	28.5 ± 0.9	24.0 ± 5.4	28.4 ± 0.0	44.3 ± 8.3	41.8 ± 2.4	55.2 ± 2.4	49.3 ± 3.5
16804594	fbaA	fructose-1,6-bisphosphate aldolase	54.1 ± 5.0	87.9 ± 4.4	87.1 ± 4.3	50.3 ± 1.0	46.8 ± 0.9	90.1 ± 0.0	97.8 ± 2.7	47.0 ± 1.4	101.6 ± 0.0	93.9 ± 0.0
16804495	tpiA	triosephosphate isomerase	32.0 ± 2.8	63.6 ± 13.1	70.4 ± 5.3	33.2 ± 0.3	33.8 ± 2.5	48.1 ± 4.5	49.2 ± 9.1	51.3 ± 6.0	54.5 ± 7.6	54.5 ± 1.5
16804497	gap	glyceraldehyde-3-phosphate dehydrogenase	151.7 ± 0.7	215.6 ± 122.5	453.8 ± 13.1	147.8 ± 1.2	151.1 ± 7.3	264.5 ± 2.2	312.9 ± 3.4	213.9 ± 6.7	203.6 ± 10.1	190.1 ± 6.7
16804496	pgk	phosphoglycerate kinase	30.1 ± 0.5	69.8 ± 13.5	88.5 ± 4.7	41.6 ± 1.5	42.0 ± 4.0	54.0 ± 4.8	66.2 ± 8.7	51.9 ± 0.0	58.0 ± 1.0	64.2 ± 5.8
16804494	pgm	phosphoglyceromutase	10.5 ± 2.2	21.2 ± 1.9	25.7 ± 2.2	13.4 ± 0.7	11.5 ± 1.2	18.5 ± 1.4	21.5 ± 1.4	19.0 ± 0.7	27.7 ± 1.4	24.1 ± 0.7
16804493	eno	phosphopyruvate hydratase	142.3 ± 1.1	325.5 ± 24.2	322.9 ± 19.0	109.6 ± 4.3	102.6 ± 6.7	237.9 ± 5.3	254.1 ± 10.5	161.1 ± 12.3	126.4 ± 5.3	128.3 ± 2.6
16803610	pykA	pyruvate kinase	25.1 ± 2.0	69.3 ± 25.3	73.6 ± 2.1	13.2 ± 0.1	11.6 ± 0.7	41.3 ± 3.9	50.1 ± 4.5	36.3 ± 1.9	32.2 ± 2.6	31.7 ± 0.6
16803092	pdhA	pyruvate dehydrogenase subunit E1 alpha	6.6 ± 1.2	20.4 ± 2.1	25.4 ± 1.7	5.5 ± 0.7	6.0 ± 0.6	15.4 ± 2.0	27.2 ± 1.0	29.3 ± 2.0	13.3 ± 1.0	20.2 ± 1.0
16803093	pdhB	pyruvate dehydrogenase subunit E1 beta	2.9 ± 1.4	21.7 ± 3.3	15.8 ± 1.7	5.0 ± 1.3	4.7 ± 0.0	10.6 ± 1.2	21.2 ± 2.3	35.0 ± 1.2	10.6 ± 1.2	11.4 ± 0.0
16803094	pdhC	dihydrolipoamide acetyltransferase E2	2.3 ± 0.8	8.0 ± 4.2	11.8 ± 2.1	3.3 ± 1.3	2.8	11.9 ± 1.4	16.3 ± 4.9	14.8 ± 1.4	9.9 ± 1.4	6.9 ± 1.4
16803095	pdhD	dihydrolipoamide dehydrogenase E3	3.4 ± 0.0	10.7 ± 1.5	11.9 ± 2.1	1.4 ± 0.0	0.7	13.4 ± 0.8	16.3 ± 1.6	12.8 ± 0.0	3.5 ± 0.0	2.3 ± 0.0
**Fermentation**												
16802256	ldh	L-lactate dehydrogenase	1.0 ± 0.0	6.5 ± 7.0	15.2 ± 5.1	3.6 ± 0.8	4.3 ± 0.7	4.2 ± 1.2	10.1 ± 2.4	4.2 ± 1.2	9.2 ± 3.6	8.4 ± 2.4
16803956	pflA	pyruvate formate-lyase	16.0 ± 2.5	26.8 ± 0.7	27.9 ± 1.0	1.7	0.4	32.6 ± 4.3	21.5 ± 5.7	20.5 ± 0.5	5.4 ± 1.9	2.0 ± 1.0
16803446	pflB	pyruvate formate-lyase	12.7 ± 1.9	35.3 ± 6.1	32.8 ± 1.5	0.4	0.0	31.9 ± 1.5	35.0 ± 1.0	26.8 ± 0.0	7.9 ± 0.5	10.3 ± 2.9
16803674	adh	acetaldehyde-CoA/alcohol dehydrogenase	41.5	70.1 ± 16.0	58.4 ± 1.2	8.2 ± 1.2	5.8 ± 0.2	77.2 ± 2.6	61.7 ± 1.3	35.6 ± 2.1	33.4 ± 3.4	34.6 ± 0.9
16804142	pta	phosphotransacetylase	9.3 ± 0.7	21.6	22.8 ± 3.2	11.7 ± 1.0	10.1 ± 0.7	16.7 ± 2.4	20.1 ± 0.0	15.9 ± 1.2	12.5 ± 1.2	13.4 ± 2.4
16803621	ackA	acetate kinase	13.1 ± 1.1	26.5 ± 6.6	30.6 ± 0.4	5.6 ± 0.1	2.6 ± 0.5	30.2 ± 1.9	28.9 ± 1.9	23.0 ± 0.9	15.1 ± 0.9	11.2 ± 2.8
**TCA**												
16803112	pycA	pyruvate carboxylase	0.7 ± 0.2	1.4 ± 0.2	3.3 ± 0.4	0.0	0.0	1.3 ± 0.6	2.0 ± 0.3	1.3 ± 0.0	1.8 ± 0.6	2.0 ± 0.3
16803681	citB	aconitate hydratase	0.0	2.1 ± 0.1	0.7 ± 0.0	0.0	0.0		0.6 ± 0.0		1.2	0.9 ± 0.4
16802400	fum	fumarate reductase subunit A	3.7 ± 1.8	26.0 ± 6.4	57.0 ± 11.5	7.7 ± 1.1	8.8 ± 0.5	4.8 ± 0.7	10.6 ± 3.0	34.4 ± 2.2	11.1 ± 2.2	11.1 ± 2.2

**PPP**												
16804017		glucose-6-phosphate 1-dehydrogenase	0.0	2.7 ± 1.1	3.5	0.0	0.0					
16803416		6-phosphogluconate dehydrogenase	12.6 ± 0.4	24.7 ± 0.8	26.8 ± 2.1	1.7 ± 0.5	0.6	20.3 ± 2.3	15.9 ± 0.8	13.7 ± 3.9	6.0 ± 0.8	3.3 ± 1.6
16803993	drm	phosphopentomutase	6.2 ± 1.1	8.2 ± 2.1	12.7 ± 0.8	4.8 ± 1.2	2.6 ± 0.5	14.5 ± 1.9	11.8 ± 0.0	9.2 ± 1.9	8.6 ± 2.8	7.2 ± 0.9
16803345	tkt	transketolase	2.8 ± 0.0	8.0 ± 4.0	11.8 ± 0.3	1.5	1.8 ± 0.6	8.0 ± 1.1	7.2 ± 1.1	3.6 ± 0.6	5.6 ± 2.3	6.8 ± 0.6
Amino sugar/UDP-Hex												
16802769	glmS	glucosamine--fructose-6-phosphate aminotransferase	1.0	1.6	6.0 ± 1.3	0.0	0.5	2.6 ± 0.0	2.6 ± 0.0	3.9 ± 0.6	7.4 ± 0.6	6.1 ± 2.5
16804157	glmM	phosphoglucosamine mutase	3.1 ± 0.5	7.4 ± 1.9	6.7 ± 0.1	1.8 ± 1.5	2.0	4.7 ± 0.0	4.7 ± 0.0	4.7 ± 1.7	8.3 ± 1.7	7.7 ± 0.8
16804149		mannnose-6 phospate isomerase	1.4 ± 0.7	1.5	1.9 ± 0.0	1.5 ± 0.7	1.9 ± 0.0	1.6 ± 0.0	1.6 ± 0.0	1.6	3.3 ± 2.3	3.3 ± 0.0
16804513		phosphoglucomutase	1.1	3.9 ± 0.9	3.6 ± 2.2	0.0	0.0	2.2 ± 0.6			3.1 ± 0.6	2.7 ± 1.3
**Etc.**												
16803127		glucitol dehydrogenase	3.1 ± 0.6	4.1	9.3 ± 3.4	1.8	0.9 ± 0.0	5.2 ± 1.1	4.5 ± 0.0	7.5 ± 0.0	6.0 ± 0.0	6.7 ± 1.1
16802237		phospho-beta-glucosidase	4.9 ± 0.0	8.4 ± 1.8	8.4 ± 1.9	3.9 ± 1.9	4.2 ± 0.9	6.3 ± 0.0	5.3 ± 1.5	3.2 ± 1.5	3.2 ± 1.5	5.3 ± 4.5
16802582		tagatose 1,6-diphosphate aldolase	2.2 ± 1.9	4.0 ± 1.7	5.2 ± 2.6	2.3 ± 1.9	1.8 ± 0.0	6.1 ± 4.3	4.6 ± 4.3	1.5 ± 0.0	2.3 ± 1.1	4.6 ± 0.0
16804034		deoxyribose-phosphate aldolase	0.0	0.0	5.5	0.0	1.4 ± 0.0					

Supplementary Table 6. The summary of protein synthesis-related protein expressions

GI	Gene	Product	Exponential					Stationary				
			10℃	20℃	30℃	37℃	42℃	10℃	20℃	30℃	37℃	42℃
**Ribosomal Protein**												
16803977	*rpsA*	30S ribosomal protein S1	2.4 ± 1.1	5.5 ± 2.3	7.9 ± 0.2	7.2 ± 0.5	3.6 ± 0.6	9.7 ± 3.9	9.7 ± 0.0	6.3 ± 2.9	15.3 ± 3.9	11.8 ± 1.0
16803698	*rpsB*	30S ribosomal protein S2	35.1 ± 1.0	182.8 ± 76.5	110.5 ± 2.4	45.1 ± 1.7	44.2 ± 0.1	63.8 ± 12.9	98.2 ± 1.4	103.3 ± 14.3	79.0 ± 5.7	73.9 ± 7.2
16804664	*rpsC*	30S ribosomal protein S3	13.8 ± 1.9	21.5 ± 9.0	23.9 ± 7.0	20.8 ± 0.6	16.2 ± 3.8	18.8 ± 0.0	31.7 ± 1.7	25.8 ± 0.0	31.7 ± 1.7	29.3 ± 1.7
16803636	*rpsD*	30S ribosomal protein S4	3.0	19.5 ± 15.4	31.6 ± 3.4	7.7 ± 2.3	7.3 ± 4.1	10.1 ± 3.6	17.7 ± 3.6	19.0 ± 1.8	15.2 ± 3.6	13.9 ± 1.8
16804653	*rpsE*	30S ribosomal protein S5	19.4 ± 8.2	56.0 ± 31.8	11.4	27.2 ± 3.7	23.7 ± 1.3	14.9 ± 2.3	14.9 ± 2.3	19.8 ± 0.0	5.0 ± 2.3	6.6 ± 0.0
16802092	*rpsF*	30S ribosomal protein S6	83.7 ± 6.6	82.8 ± 38.8	39.8 ± 0.9	131.0 ± 14.7	90.5 ± 1.8	50.0 ± 0.0	50.0 ± 0.0	35.0 ± 7.1	47.5 ± 10.6	72.5 ± 10.6
16804692	*rpsG*	30S ribosomal protein S7	55.0 ± 2.5	38.1 ± 15.9	44.1 ± 1.0	54.2 ± 3.1	39.0 ± 2.5	58.1 ± 18.3	61.4 ± 9.1	9.7 ± 0.0	50.1 ± 2.3	63.0 ± 6.9
16804656	*rpsH*	30S ribosomal protein S8	46.3 ± 3.1	22.7 ± 3.8	17.9 ± 5.9	52.9 ± 7.8	34.0 ± 0.1	23.6 ± 0.0	23.6 ± 5.6	17.7 ± 2.8	25.6 ± 2.8	27.6 ± 0.0
16804634	*rpsI*	30S ribosomal protein S9	35.2 ± 0.1	18.4	47.7 ± 2.2	40.2 ± 0.9	33.3 ± 1.5	33.9 ± 2.8	37.9 ± 2.8	20.0 ± 0.0	31.9 ± 0.0	41.9 ± 8.5
16804671	*rpsJ*	30S ribosomal protein S10	109.7 ± 8.6	120.0 ± 54.3	103.5 ± 6.2	100.5 ± 12.6	87.6 ± 0.3	108.1 ± 7.0	86.0 ± 10.4	88.5 ± 20.9	90.9 ± 10.4	100.8 ± 17.4
16804645	*rpsK*	30S ribosomal protein S11	58.8 ± 3.7	112.9 ± 40.2	113.8 ± 9.2	87.6 ± 3.7	69.5 ± 3.2	75.0 ± 11.8	97.9 ± 2.9	81.3 ± 8.8	79.2 ± 5.9	85.4 ± 14.7
16803370	*rpsO*	30S ribosomal protein S15	3.2 ± 0.0	9.2	9.3 ± 4.6	6.6 ± 0.1	4.7 ± 2.2				5.4	
16803837	*rpsP*	30S ribosomal protein S16	19.5 ± 0.1	31.5 ± 8.5	22.0 ± 4.0	20.2 ± 0.4	19.1 ± 4.6	33.2 ± 0.0	22.1 ± 0.0	19.4 ± 3.9	22.1 ± 7.8	30.4 ± 3.9
16804661	*rpsQ*	30S ribosomal protein S17	47.3 ± 0.2	37.1	26.2 ± 0.6	40.2 ± 16.6	74.4 ± 2.6	28.8 ± 0.0	8.6 ± 4.1	23.0 ± 16.3		
16804666	*rpsS*	30S ribosomal protein S19	57.9 ± 4.8	40.4	40.6 ± 14.1	93.5 ± 2.9	91.3 ± 4.7	54.8 ± 0.0	24.6 ± 3.9	19.2 ± 3.9	24.6 ± 3.9	24.6 ± 3.9
16802295	*rplA*	50S ribosomal protein L1	30.3 ± 4.0	169.7 ± 80.9	80.0 ± 2.0	47.3 ± 3.0	58.0 ± 1.7	43.4 ± 5.0	70.4 ± 3.3	76.3 ± 1.7	57.5 ± 5.0	41.1 ± 11.6
16804667	*rplB*	50S ribosomal protein L2	4.4 ± 1.5	4.9 ± 2.1	20.3 ± 7.1	0.0	0.0	4.7 ± 4.0	4.7 ± 1.3	4.7 ± 1.3	5.7	
16804670	*rplC*	50S ribosomal protein L3	25.9 ± 3.2	118.4 ± 10.1	83.2 ± 5.9	43.9 ± 2.0	50.0 ± 3.2	26.5 ± 1.8	69.4 ± 8.9	60.5 ± 7.1	41.6 ± 5.4	47.9 ± 10.7
16804669	*rplD*	50S ribosomal protein L4	18.7 ± 5.4	97.3 ± 14.9	94.1 ± 8.2	18.7 ± 4.8	23.4 ± 0.1	38.2 ± 7.2	48.3 ± 7.2	63.6 ± 3.6	36.9 ± 1.8	34.3 ± 5.4
16804658	*rplE*	50S ribosomal protein L5	60.8 ± 5.0	166.4 ± 40.5	135.7 ± 0.6	58.7 ± 2.6	52.1 ± 3.7	67.6 ± 10.2	96.3 ± 6.1	138.0 ± 20.3	48.9 ± 16.3	57.5 ± 0.0
16804655	*rplF*	50S ribosomal protein L6	80.1 ± 2.8	177.3 ± 69.2	124.7 ± 2.1	81.3 ± 1.0	76.7 ± 4.6	94.9 ± 4.2	108.2 ± 6.3	111.2 ± 10.5	100.8 ± 8.4	109.7 ± 8.4
16802297	*rplL*	50S ribosomal protein L7/L12	173.0 ± 19.8	602.7 ± 664.8	1206 ± 22.0	147.2 ± 15.0	134.9 ± 15.4	294.4 ± 110.6	524.4 ± 19.5	823.4 ± 13.0	218.5 ± 9.8	149.5 ± 9.8
16802101	*rplI*	50S ribosomal protein L9	17.7 ± 1.4	3.3	20.2 ± 6.1	44.4 ± 0.7	35.7 ± 4.4	21.3 ± 0.0	16.0 ± 2.5	5.3 ± 2.5	37.3 ± 7.5	40.8 ± 2.5
16802296	*rplJ*	50S ribosomal protein L10	37.2 ± 1.5	115.0 ± 39.9	57.4 ± 9.1	33.7 ± 3.5	31.7 ± 2.7	39.0 ± 4.6	40.6 ± 16.1	35.7 ± 4.6	30.9 ± 2.3	32.5 ± 4.6
16802294	*rplK*	50S ribosomal protein L11	47.6 ± 0.2	81.8	85.6 ± 1.2	62.4 ± 2.9	63.2 ± 10.8	46.3 ± 10.9	69.5 ± 5.5	28.9 ± 2.7	40.5 ± 8.2	52.1 ± 8.2
16804635	*rplM*	50S ribosomal protein L13	49.0 ± 4.6	60.7 ± 16.4	30.2 ± 2.2	61.7 ± 0.3	62.2 ± 4.1	83.4 ± 2.5	58.6 ± 2.5	24.8 ± 0.0	67.4 ± 5.0	81.6 ± 5.0
16804651	*rplO*	50S ribosomal protein L15	24.6 ± 1.6	34.4 ± 22.5	53.8 ± 1.2	37.7 ± 2.4	30.3 ± 1.6	36.4 ± 0.0	52.8 ± 2.6	38.2 ± 7.7	38.2 ± 2.6	34.6 ± 2.6
16804663	*rplP*	50S ribosomal protein L16	9.4 ± 1.4	20.3 ± 5.5	26.4 ± 2.3	6.5 ± 0.1	8.2 ± 2.9	12.5 ± 2.5	12.5 ± 2.5	25.0 ± 5.1	12.5 ± 2.5	10.7 ± 0.0
16804643	*rplQ*	50S ribosomal protein L17	11.1 ± 3.1	20.6 ± 20.1	38.7 ± 5.2	10.4 ± 1.8	19.6 ± 3.1	20.8 ± 2.7	22.7 ± 5.4	28.4 ± 2.7	24.6 ± 2.7	17.0 ± 2.7

16804654	*rplR*	50S ribosomal protein L18	23.2 ± 0.1	32.4	37.5 ± 4.3	33.5 ± 1.2	34.1 ± 1.7	26.3 ± 6.2	41.7 ± 9.3	19.8 ± 9.3	13.2 ± 0.0	24.1 ± 3.1
16803827	*rplS*	50S ribosomal protein L19	0.0	24.7 ± 17.7	12.4 ± 10.3	6.7 ± 2.0	6.3 ± 1.8			17.6 ± 6.2	4.4	4.4
16803582	*rplU*	50S ribosomal protein L21	138.7 ± 8.0	345.4 ± 127.1	239.2 ± 11.3	222.3 ± 4.5	218.4 ± 4.8	174.6 ± 14.5	243.9 ± 32.7	151.5 ± 18.2	213.1 ± 3.6	195.1 ± 36.3
16804665	*rplV*	50S ribosomal protein L22	27.5 ± 9.4	60.1 ± 32.7	38.1 ± 4.4	39.4 ± 2.7	34.6 ± 5.3	22.3 ± 0.0	26.7 ± 0.0	51.3 ± 3.2	24.5 ± 3.2	15.6 ± 3.2
16804668	*rplW*	50S ribosomal protein L23	43.4 ± 4.2	106.4 ± 26.7	66.0 ± 1.4	56.4 ± 12.5	66.7 ± 0.2	52.8 ± 14.9	68.6 ± 7.5	58.0 ± 7.5	50.1 ± 3.7	39.6 ± 3.7
16804659	*rplX*	50S ribosomal protein L24	25.7 ± 6.5	35.6 ± 23.6	31.9 ± 20.0	31.3 ± 3.8	38.4 ± 8.2	20.5 ± 0.0	25.7 ± 7.3	10.3		20.5
16802257	*ctc*	50S ribosomal protein L25	14.9 ± 0.1	12.1 ± 7.2	14.4 ± 7.9	17.1 ± 0.3	21.2 ± 1.1	24.2 ± 1.8	25.4 ± 0.0	7.6	31.8 ± 1.8	34.3 ± 1.8
16803580	*rpmA*	50S ribosomal protein L27	69.1 ± 11.1	96.1 ± 21.7	81.0 ± 1.8	85.1 ± 10.1	80.3 ± 2.5	82.1 ± 0.0	49.3 ± 7.7	43.8 ± 7.7	43.8 ± 15.5	43.8
16804662	*rpmC*	50S ribosomal protein L29	89.0 ± 2.9	111.9 ± 46.8	79.7 ± 14.2	82.9 ± 8.4	62.5 ± 0.2	101.0 ± 0.0	73.8 ± 16.5	108.8 ± 11.0	73.8 ± 5.5	97.1 ± 5.5
16804586	*rpmE2*	50S ribosomal protein L31	67.2 ± 2.3	48.6 ± 20.3	52.7 ± 3.8	94.3 ± 1.9	81.7 ± 4.8	80.4 ± 0.0	74.2 ± 0.0	6.2 ± 0.0	71.1 ± 4.4	80.4 ± 8.7
**t-RNA synthetase**												
16802223	*metS*	methionyl-tRNA synthetase	0.9	4.2	11.7 ± 1.6	0.5	0.0	3.0 ± 1.1	2.7 ± 0.5	6.1 ± 0.0	10.6 ± 3.2	8.7 ± 0.5
16802274	*lysS*	lysyl-tRNA synthetase	3.8 ± 1.3	7.9 ± 3.3	11.4 ± 0.2	3.4 ± 1.4	3.2 ± 2.0	8.5 ± 3.5	8.5 ± 0.7	5.5 ± 2.1	16.5 ± 0.7	17.0 ± 4.3
16802283	*gltX*	glutamyl-tRNA synthetase	0.6	4.0 ± 1.7	7.0 ± 1.8	1.6 ± 0.5	0.3	2.1	2.1	2.6 ± 0.7	2.1 ± 0.0	3.1
16803262	*pheT*	phenylalanyl-tRNA synthetase subunit beta	0.0	1.8	3.0	0.0	0.0		1.3	1.6 ± 0.5	0.7	
16803498	*glyS*	glycyl-tRNA synthetase subunit beta	0.0	2.7	1.7 ± 0.0	0.0	0.0			2.2		
16803544	*alaS*	alanyl-tRNA synthetase	0.0	5.7 ± 0.4	8.4 ± 1.6	0.4	0.0		2.6 ± 1.2	7.0 ± 0.8	5.0 ± 0.4	5.6 ± 0.4
16803559	*aspS*	aspartyl-tRNA synthetase	1.0 ± 0.7	4.9 ± 1.3	6.4 ± 0.6	0.0	0.0	1.7	2.2 ± 0.6	3.9 ± 0.6	4.3	1.7 ± 0.0
16803647	*pheT*	phenylalanyl-tRNA synthetase subunit beta	3.0	14.6 ± 3.9	7.3 ± 1.9	6.2 ± 2.1	5.2 ± 1.0		7.7 ± 3.6	11.6 ± 1.8	7.7 ± 0.0	5.1 ± 0.0
16803794	*gatB*	aspartyl/glutamyl-tRNA amidotransferase subunit B	3.5 ± 1.3	23.6 ± 8.1	28.9 ± 1.5	5.6 ± 0.6	5.9 ± 2.2	4.3 ± 3.1	10.8 ± 0.0	25.4 ± 3.8	16.8 ± 3.8	16.8 ± 5.4
16803795	*gatA*	aspartyl/glutamyl-tRNA amidotransferase subunit A	2.9 ± 0.5	4.8 ± 1.1	12.5 ± 0.3	3.3 ± 0.9	1.3	7.1 ± 0.8	7.1 ± 2.3	4.9 ± 0.8	13.7 ± 2.3	10.4 ± 0.8
16804058	*ileS*	isoleucyl-tRNA synthetase	0.3	2.0 ± 0.1	2.8 ± 0.5	0.0	0.2	1.4 ± 0.4	1.1 ± 0.0	1.7 ± 0.8	2.5 ± 0.4	3.6 ± 0.4
16804599	*argS*	arginyl-tRNA synthetase	1.1	3.4	4.2 ± 1.6	0.0	0.0		6.4	0.9	3.7 ± 0.0	6.9 ± 3.2
**DNA/RNA polymerase**												
16802050	*dnaN*	DNA polymerase III subunit beta	1.6 ± 0.0	1.3	3.8 ± 1.0	1.7	1.2 ± 0.6	3.4 ± 1.0	4.7 ± 1.0	3.4 ± 1.0	4.7 ± 1.0	4.7 ± 1.0
16804644	*rpoA*	DNA-directed RNA polymerase subunit alpha	2.9 ± 1.4	7.4 ± 2.5	10.3 ± 1.5	4.0 ± 0.1	4.3 ± 0.7	4.1 ± 3.5	8.2 ± 2.3	7.4 ± 1.2	8.2 ± 2.3	12.4 ± 1.2
16802304	*rpoB*	DNA-directed RNA polymerase subunit beta	3.2 ± 0.9	7.7 ± 0.5	11.4 ± 0.2	0.8 ± 0.4	0.6 ± 0.2	8.9 ± 0.3	6.9 ± 0.6	6.5 ± 0.6	5.2 ± 2.5	7.8 ± 0.0
16802305	*rpoC*	DNA-directed RNA polymerase subunit beta'	5.0 ± 1.0	17.8 ± 3.5	20.4 ± 0.9	1.7 ± 0.6	1.0 ± 0.3	10.9 ± 1.5	13.7 ± 1.2	11.5 ± 1.8	10.5 ± 0.3	11.7 ± 0.9

16804598	*rpoZ*	DNA-directed RNA polymerase subunit delta	4.1 ± 1.1	6.2 ± 2.1	11.2 ± 7.0	5.9 ± 1.1	4.0 ± 1.1	8.4 ± 0.0	12.6 ± 2.0	2.8	8.4 ± 3.9	11.2 ± 0.0
16803866	*rpoE*	DNA-directed RNA polymerase subunit omega	4.4 ± 0.0	0.0	0.0	4.7	8.7					
**Helicase**												
16803799	*pcrA*	ATP-dependent DNA helicase	0.0	0.0	0.0	0.0	0.0				1.4	1.4
16802907		ATP-dependent RNA helicase	15.9 ± 1.7	39.6 ± 8.9	31.9 ± 0.9	2.7 ± 1.2	2.6 ± 2.0	41.6 ± 2.1	36.1 ± 4.3	23.6 ± 0.7	22.1 ± 0.0	16.6 ± 2.1
16803490		DEAD/DEAH box helicase	2.4 ± 1.4	1.1	0.0	0.0	0.0	7.0 ± 3.3	2.3 ± 0.0		1.2	
**Cofactors**												
16803362	*nusA*	transcription elongation factor NusA	8.1 ± 0.0	18.5 ± 6.3	19.6 ± 1.5	6.7 ± 1.1	4.4 ± 1.7	15.9 ± 2.9	16.5 ± 0.0	15.9 ± 1.0	13.8 ± 0.0	13.1 ± 2.9
16803536	*greA*	transcription elongation factor GreA	16.4 ± 1.3	11.4 ± 7.5	7.6	25.1 ± 1.9	9.4 ± 5.3	16.4 ± 0.0	16.4 ± 0.0		23.0	27.9 ± 7.0
16804648	*infA*	translation initiation factor IF-1	18.5 ± 2.8	15.1 ± 12.2	31.9 ± 0.7	29.9 ± 5.5	32.2 ± 0.1	24.5 ± 5.0	24.5 ± 5.0	7.0	24.5 ± 5.0	21.0 ± 9.9
16803365	*infB*	translation initiation factor IF-2	1.2 ± 0.6	3.0 ± 0.7	4.3 ± 0.6	0.4 ± 0.0	0.2	3.4 ± 1.0	5.1 ± 0.5	2.7 ± 1.0	5.1 ± 0.5	5.4 ± 1.9
16803825	*infC*	translation initiation factor IF-3	10.3 ± 0.0	20.3 ± 13.5	13.3 ± 0.3	12.6 ± 2.8	11.8 ± 0.0	7.3 ± 2.1	11.7 ± 8.3	8.8 ± 0.0	13.2 ± 2.1	14.7 ± 4.1
16803697	*tsf*	elongation factor Ts	34.2 ± 1.6	63.8 ± 2.8	74.2 ± 1.6	70.0 ± 4.4	71.0 ± 1.2	52.9 ± 5.0	75.8 ± 2.5	57.3 ± 1.2	83.8 ± 11.2	100.5 ± 5.0
16804690	*tuf*	elongation factor Tu	75.7 ± 3.6	295.1 ± 181.6	539.3 ± 18.1	115.5 ± 6.3	124.4 ± 4.7	164.7 ± 15.0	282.9 ± 1.9	523.2 ± 22.5	411.0 ± 40.4	333.3 ± 7.5
16804691	*fus*	elongation factor G	14.1 ± 1.3	64.3 ± 15.5	80.8 ± 5.5	9.6 ± 2.8	6.9 ± 1.2	25.5 ± 3.2	36.7 ± 1.1	55.4 ± 4.2	41.6 ± 4.8	41.9 ± 4.2
16803107		GTP-binding elongation factor	5.9 ± 0.7	9.2 ± 1.0	9.5 ± 1.6	0.5	0.2	10.9 ± 0.0	9.2 ± 1.2	8.4 ± 2.4	5.0 ± 0.0	3.3 ± 0.0
16804581	*prfA*	peptide chain release factor 1	0.8	0.0	0.0	4.3 ± 0.1	2.4 ± 0.0			1.4	4.2 ± 2.0	2.8 ± 2.0
16803091	*def*	peptide deformylase	0.0	0.0	3.2	10.3 ± 5.0	6.4 ± 4.5				5.6 ± 0.0	8.4
16802259	*pth*	peptidyl-tRNA hydrolase	0.0	0.0	0.0	1.7 ± 0.0	1.6				2.7	
**Etc**												
16803354	*frr*	ribosome recycling factor	35.9 ± 0.1	6.2 ± 2.1	6.2	42.4 ± 3.2	31.1 ± 3.3	34.7 ± 2.0	44.4 ± 3.9		44.4 ± 0.0	54.2 ± 2.0
16803511	*prmA*	ribosomal protein L11 methyltransferase	0.0	0.0	1.9	0.0	0.0	3.3 ± 0.0	1.7		1.7	

Supplementary Table 7. The summary of the transport-related proteins expressions

GI	Gene	Product	Exponential					Stationary				
			10℃	20℃	30℃	37℃	42℃	10℃	20℃	30℃	37℃	42℃
**PTS type**												
16802144		PTS mannose transporter subunit IIAB	12.1 ± 0.7	23.8 ± 5.8	19.6 ± 3.5	16.5 ± 1.0	12.3 ± 0.0	20.5 ± 1.2	28.8 ± 1.2	11.5 ± 4.6	15.6 ± 1.2	23.8 ± 5.8
16802146		PTS mannose transporter subunit IID	12.6 ± 0.8	40.7 ± 12.7	30.3 ± 0.7	4.2 ± 0.1	4.9 ± 0.0	23.2 ± 1.2	27.5 ± 2.4	26.7 ± 1.2	24.1 ± 0.0	24.1 ± 2.4
16803057		PTS glucose transporter subunit IIA	4.8 ± 1.3	0.0	0.0	6.9 ± 1.3	4.7 ± 1.3		6.5		8.1 ± 2.3	6.5 ± 4.6
16803135		PTS cellbiose transporter subunit IIB	0.0	0.0	0.0	2.4	2.3					
16804411		PTS beta-glucoside transporter subunit IIB	42.2 ± 8.4	36.0 ± 10.7	37.9 ± 3.3	28.2 ± 5.0	28.0 ± 2.0	48.8 ± 3.6	43.6 ± 10.9	41.1 ± 0.0	41.1 ± 7.3	38.5 ± 3.6
16804720		PTS cellbiose transporter subunit IIB	10.9 ± 2.2	0.0	0.0	3.3	0.0	23.7 ± 3.7				
16803043		phosphotransferase system enzyme I	12.8 ± 0.8	25.8 ± 1.5	33.1 ± 2.2	7.0 ± 2.1	5.2 ± 0.0	28.7 ± 1.9	28.7 ± 3.2	16.4 ± 1.3	28.7 ± 4.5	29.6 ± 3.2
**ABC type**												
16802183		peptide ABC transporter substrate-binding protein	2.0 ± 0.4	12.9 ± 5.4	17.9 ± 2.8	2.1 ± 0.5	2.0 ± 1.2	3.9	8.4 ± 4.9	9.9 ± 0.0	2.5 ± 0.7	4.4 ± 2.1
16803054		glycine/betaine ABC transporter ATP-binding protein	1.9 ± 0.6	8.5	8.2 ± 0.9	1.6 ± 1.1	1.5 ± 0.0	7.3 ± 0.9	7.3 ± 2.8	4.0 ± 0.0	4.0	4.6 ± 2.8
16804153		ABC transporter ATP-binding protein	3.6 ± 1.7	15.0	12.7 ± 1.4	8.0 ± 0.7	4.7 ± 1.6	11.2 ± 4.3	18.3 ± 0.0	5.1 ± 4.3	25.4 ± 7.2	23.4 ± 1.4
16804232		peptide ABC transporter ATP-binding protein	1.7	6.7	6.6 ± 2.5	1.8 ± 0.0	1.2 ± 0.6	2.9	3.6 ± 1.0	7.2 ± 2.0	2.9	2.2 ± 1.0
16804453		ABC transporter ATP-binding protein	5.8 ± 1.7	17.6 ± 6.0	22.4 ± 5.9	9.0 ± 0.7	6.8 ± 0.0	9.9 ± 0.0	20.7 ± 1.4	25.7 ± 11.2	24.7 ± 4.2	22.7 ± 7.0
**Etc**												
16804398		transmembrane protein	2.4 ± 0.0	2.6 ± 0.6	5.0 ± 0.4	0.5 ± 0.3	0.9 ± 0.2		1.5 ± 0.4	1.2 ± 0.0		

Supplementary Table 8. The summary of protease expressions

GI	Gene	Product	Exponential					Stationary				
			10℃	20℃	30℃	37℃	42℃	10℃	20℃	30℃	37℃	42℃
16803651		aminopeptidase	2.2±0.6	4.1	5.1±0.1	3.6±0.1	3.0±1.8	4.4±0.0	7.4±2.1	4.4±2.1	4.4±0.0	5.2±1.0
16803751		aminopeptidase	6.0±0.0	14.7±0.7	15.3±0.7	1.9±0.5	1.8±0.5	11.5±0.0	11.5±3.6	10.9±0.9	10.2±3.6	8.3±0.9
16803394		aminopeptidase P	0.0	3.9±1.6	5.9±1.3	0.0	0.0	2.2±1.0	6.6±3.1	3.7±3.1		
16803820		aminotripeptidase	1.5±0.0	4.4±2.9	2.1±1.0	0.8	1.1±0.5	3.8	3.8±1.8	1.3	3.8±0.0	3.8±1.8
16804245	clpB	Clp protease subunit B	0.0	1.5±0.6	3.7±0.4	4.9±0.2	6.1±0.5			4.4±1.3	20.3±0.4	26.0±1.7
16802278	clpC	endopeptidase Clp ATP-binding chain C	0.0	2.3	2.2±0.0	0.4±0.0	0.0	1.9±0.9	0.9±0.4	4.4±0.9	7.3±1.3	6.6±1.3
16803178	clpP	ATP-dependent Clp protease proteolytic subunit	1.6±0.0	3.5±1.5	6.0	14.0±3.8	12.4±0.0	2.7±0.0	2.7±0.0	6.7±1.9	25.6±1.9	27.0±0.0
16804506	clpP	ATP-dependent Clp protease proteolytic subunit	3.1	4.9	16.6±6.1	8.9±1.0	10.7±2.2	9.3±1.9	8.0±3.8	5.3	16.0±3.8	12.0±1.9
16803308	clpX	ATP-dependent protease ATP-binding subunit ClpX	0.7	12.6	10.6±1.2	0.8±0.0	0.0	3.1±0.9	6.2±0.0	6.8±0.9	8.1±4.4	8.1±4.4
16803660	pepV	dipeptidase PepV	3.6±0.5	16.3±11.5	11.3±5.1	1.0±0.5	2.9±0.5	7.8±0.0	9.4±2.4	8.3±2.4	7.2±0.8	5.6±0.0
16802625		invasion associated secreted endopeptidase	0.7	0.0	0.0	0.0	0.3				1.1	3.4±1.6
16803749		methionine aminopeptidase	1.8±0.9	14.4±4.3	11.7±0.3	6.3±0.1	5.9±1.7	3.1±1.5	7.2±1.5	12.4±0.0	10.3±0.0	12.4±2.9
16803001		protease	0.0	0.0	0.0	0.0	0.0				1.3	1.3
16803618		X-Pro dipeptidase	1.7	2.6	5.7±1.0	0.9±0.0	1.2±0.6	4.3±2.0	7.1	5.0±3.0	2.9±0.0	4.3
16803257		Glytamyl aminopeptidase	0.0	0.0	2.5±1.1	0.0	0.0	3.0±0.0	2.2±1.0	1.5±0.0		1.5

Supplementary Table 9. The summary of functional protein expressions

GI	Gene	Product	Exponential					Stationary				
			10℃	20℃	30℃	37℃	42℃	10℃	20℃	30℃	37℃	42℃
**Motility**												
16803739	cheV	chemotaxis protein	11.3 ± 1.4	4.8	0.0	0.0	0.0	20.0 ± 3.7	6.1 ± 1.2			
16804059	cheY	chemotaxis protein CheY	12.5 ± 3.6	17.4 ± 2.5	16.1 ± 4.2	19.9 ± 0.8	14.7 ± 2.3	17.0 ± 4.0	11.3 ± 4.0	12.7 ± 2.0	24.1 ± 2.0	25.5 ± 4.0
16802732	flaA	flagellin	300.6 ± 0.4	88.5 ± 1.9	46.3 ± 5.6	0.0	0.0	323.5 ± 2.7	81.3 ± 2.7	27.4 ± 6.7		
16802739	flgE	flagellar hook protein FlgE	9.1 ± 0.6	5.3 ± 2.2	0.0	0.0	0.0	14.8 ± 0.0	8.7 ± 1.0			
16804330		major tail shaft protein	18.3 ± 1.6	7.9 ± 6.4	8.3 ± 0.2	0.0	0.0	23.8 ± 2.6	20.1 ± 2.6	12.8 ± 7.8		
**REDOX**												
16804516	trxB	thioredoxin reductase	0.0	4.6	3.8 ± 0.1	0.0	2.9		5.0	3.4	6.7 ± 2.4	10.1 ± 2.4
16804191	trxA	thioredoxin	13.6 ± 3.8	18.2 ± 7.6	10.7	14.1 ± 3.7	8.0 ± 3.8	4.6 ± 0.0	18.5		11.6 ± 3.3	9.3
16803273	trxA	thioredoxin	17.5 ± 0.1	17.5 ± 1.0	39.5 ± 0.9	21.2 ± 0.4	21.4 ± 2.0	37.2 ± 3.5	37.2 ± 3.5	29.7 ± 0.0	44.6 ± 0.0	49.6 ± 7.0
16803644		2-cys peroxiredoxin	1.7 ± 0.0	7.5 ± 3.1	6.5 ± 0.1	2.6 ± 1.2	2.5 ± 1.2		2.9 ± 0.0	4.3 ± 2.0	4.3 ± 2.0	7.2 ± 2.0
16804676		NADH dehydrogenase	6.2 ± 2.0	15.0 ± 7.5	23.7 ± 0.1	5.5 ± 0.8	4.9 ± 1.0	12.3 ± 0.0	16.3 ± 1.2	19.2 ± 0.6	14.7 ± 1.2	15.5 ± 1.2
16804427		NADH dehydrogenase	0.0	1.2	3.7 ± 1.1	0.0	1.5	2.6	1.3	5.9 ± 0.9	2.6 ± 0.0	4.6 ± 0.9
16804509		NADPH dehydrogenase	0.9 ± 0.0	2.0 ± 0.9	3.5 ± 0.1	1.9	1.3 ± 0.6	3.9 ± 1.1	2.3 ± 1.1			
16802400		fumarate reductase subunit A	3.7 ± 1.8	26.0 ± 6.4	57.0 ± 11.5	7.7 ± 1.1	8.8 ± 0.5	4.8 ± 0.7	10.6 ± 3.0	34.4 ± 2.2	11.1 ± 2.2	11.1 ± 2.2
**Protein Translocation**												
16804548	secA	preprotein translocase subunit SecA	2.1 ± 0.5	2.1 ± 1.4	4.8 ± 2.8	0.0	0.0	4.0 ± 1.3	4.0 ± 0.4	3.6 ± 0.9	4.0 ± 1.3	2.7 ± 0.4
16803567	secDF	preprotein translocase SecDF	0.0	0.6	1.6 ± 0.0	0.0	0.0			1.4		
16804489	secG	preprotein translocase subunit SecG	0.0	9.3 ± 3.9	8.1 ± 0.2	0.0	0.0				7.1	
**Secondary Metabolite**												
16804602		4-oxalocrotonate isomerase	14.7 ± 0.1	0.0	19.0 ± 0.4	12.7 ± 3.3	7.2	8.3	16.7	8.3 ± 0.0		
16803640	aroA	3-deoxy-7-phosphoheptulonate synthase/chorismate mutase	4.3 ± 1.2	4.6 ± 3.0	5.8 ± 1.3	1.8 ± 1.3	1.7 ± 0.0	6.5 ± 3.1	11.6 ± 2.0	5.1 ± 1.0	5.1 ± 1.0	6.5 ± 1.0
16804452	menB	naphthoate synthase	2.8 ± 0.0	7.5 ± 4.9	8.1 ± 3.7	5.5 ± 0.6	4.8 ± 0.0	7.2 ± 1.7	11.4 ± 2.5	9.6 ± 0.0	6.0 ± 1.7	9.6 ± 3.4
16803713	menB	naphthoate synthase	5.1 ± 0.8	12.5 ± 5.2	9.8 ± 1.3	1.7 ± 0.8	0.5	10.5 ± 1.4	7.7 ± 2.7	6.7 ± 1.4	4.8 ± 1.4	2.9 ± 1.4
**Signaling / Regulator**												
16803639	ccpA	catabolite control protein A	3.6	28.7 ± 4.1	21.3 ± 8.0	4.8 ± 1.4	4.5 ± 0.0	11.7 ± 1.1	17.9 ± 3.3	15.5 ± 4.4	9.3 ± 2.2	6.2
16803042	ptsH	ptsH:p, phosphocarrier protein HPr	384.6 ± 32.0	564.6 ± 184.5	250.5 ± 5.4	568.8 ± 17.8	589.0 ± 24.1	238.6 ± 17.3	214.1 ± 8.7	165.2 ± 17.3	183.5 ± 8.7	211.0 ± 38.9
16802932	rsbT	positive regulation of sigma-B activity	12.6 ± 1.7	10.2 ± 4.3	6.6 ± 3.0	15.5 ± 4.8	12.4 ± 1.6	15.6 ± 5.5	3.9 ± 0.0		3.9	5.9 ± 2.8
16802243	spoVG	regulatory protein SpoVG	146.7 ± 1.5	264.1 ± 110.4	172.4 ± 28.1	178.5 ± 7.9	152.2 ± 2.5	214.4 ± 32.1	216.9 ± 7.1	181.6 ± 0.0	216.9 ± 7.1	234.6 ± 17.8

16802242	spoVG	regulatory protein SpoVG	24.1 ± 0.1	36.0 ± 10.7	11.7 ± 0.3	34.4 ± 3.8	28.0 ± 2.0	23.1 ± 3.6	20.5 ± 0.0	10.3	33.4 ± 10.9	28.2 ± 3.6
16803320	codY	transcriptional repressor CodY	6.5 ± 0.9	0.0	9.1 ± 0.2	6.7 ± 0.7	5.2 ± 0.8	8.0 ± 0.0	12.0 ± 2.8	7.0 ± 1.4	11.0 ± 1.4	9.0 ± 1.4
16803841	ffh	signal recognition particle protein Ffh	0.7	4.3	4.0 ± 1.9	1.4 ± 1.0	1.3	1.2	1.7 ± 0.8	1.2	5.2 ± 0.8	5.8 ± 1.6
16802935	rsbW	serine-protein kinase RsbW	7.7 ± 0.0	9.1	16.9 ± 8.3	6.1	4.7 ± 1.3	9.9 ± 4.6	11.5 ± 2.3	9.9 ± 0.0	6.6 ± 4.6	13.1
16803976	engA	GTP-binding protein EngA	2.8 ± 1.0	3.2	2.6 ± 1.8	0.0	0.0	4.1 ± 0.8	5.3 ± 2.5	1.8 ± 0.8	7.0 ± 1.7	3.5
**Nucleotide metabolism**												
16803564	apt	adenine phosphoribosyltransferase	12.3 ± 2.5	18.4 ± 2.7	13.7 ± 5.1	3.7 ± 0.1	3.5	21.0 ± 0.0	19.5 ± 6.4	10.5 ± 6.4	6.0 ± 0.0	7.5 ± 2.1
16804649	adk	adenylate kinase	7.0 ± 0.0	0.0	18.9 ± 3.4	18.9 ± 2.4	14.3 ± 2.9	8.3 ± 1.7	13.1 ± 5.0	4.8	15.4 ± 1.7	16.6 ± 0.0
16804576	upp,	uracil phosphoribosyltransferase	16.2 ± 6.3	30.9 ± 4.4	34.2 ± 3.3	19.1 ± 2.9	18.0 ± 3.0	23.9 ± 1.8	25.1 ± 7.1	26.4 ± 1.8	21.3 ± 8.9	22.6 ± 0.0
16803896	pnp	purine nucleoside phosphorylase	6.7 ± 1.9	16.3 ± 6.6	16.8 ± 2.2	4.1	2.6 ± 0.0	13.6 ± 0.0	18.2 ± 3.2	10.2 ± 1.6	4.5	
16803992	deoD	purine nucleoside phosphorylase	3.4	10.2 ± 4.3	4.5	1.2 ± 0.0	1.1	2.9 ± 1.4	3.9	1.9		
16802704	thiD	phosphomethylpyrimidine kinase	8.2	12.1 ± 6.8	10.2 ± 1.8	4.9 ± 1.8	4.6 ± 1.6	3.0 ± 1.4	9.0 ± 1.4	9.0 ± 4.2	7.0 ± 1.4	6.0 ± 2.8
16803371	pnpA	polynucleotide phosphorylase	3.2 ± 1.5	11.3 ± 4.7	7.0 ± 1.9	1.1 ± 0.3	1.0 ± 0.3	6.1 ± 0.5	6.1 ± 1.5	3.3 ± 0.5	4.7 ± 1.5	5.4 ± 0.5
16804795	guaB	inosine-monophosphate dehydrogenase	4.2 ± 1.4	7.9 ± 5.9	18.7 ± 0.4	9.0 ± 0.3	11.0 ± 1.4	6.0 ± 0.8	11.5 ± 2.3	17.0 ± 0.8	20.3 ± 0.8	20.3 ± 2.3
16804526	uvrA	excinuclease ABC subunit A	0.0	1.0	2.8 ± 1.2	1.0	0.0	0.5		1.1 ± 0.8	3.5 ± 1.1	8.1 ± 0.8
16804527	uvrB	excinuclease ABC subunit B	0.0	0.0	0.0	0.0	0.0				3.0 ± 1.1	
16804194		ribonucleotide-diphosphate reductase subunit alpha	0.8 ± 0.6	2.9 ± 1.0	9.4 ± 0.3	0.8 ± 0.0	0.2	3.3 ± 0.9	3.3 ± 0.9	7.2 ± 0.9	5.3 ± 0.0	4.0 ± 0.9
16804193	nrdF	ribonucleotide-diphosphate reductase subunit beta	2.5	1.3	22.0 ± 1.6	3.5 ± 1.3	0.0	3.6 ± 1.0	5.7 ± 0.0	9.3 ± 1.0	19.3 ± 3.0	29.3 ± 7.1
16802245	prs	ribose-phosphate pyrophosphokinase	7.2 ± 0.7	22.9 ± 11.0	23.4 ± 4.5	1.0	2.8 ± 1.3	13.1 ± 0.0	15.6 ± 3.5	15.6 ± 3.5	13.1 ± 2.3	15.6 ± 1.2
16802325		anaerobic ribonucleoside triphosphate reductase	3.9 ± 0.9	4.0 ± 1.1	9.6 ± 2.1	0.0	0.0	6.4 ± 0.0	5.7 ± 1.0	4.6 ± 1.5	2.1 ± 0.0	2.5 ± 0.5
**Cell Wall structure**												
16803012	dltC	D-alanine--poly(phosphoribitol) ligase subunit 2	24.4 ± 2.6	29.4	29.1 ± 0.6	31.2 ± 6.1	29.4 ± 0.1	12.8 ± 0.0	31.9 ± 0.0	12.8 ± 0.0	19.2 ± 9.0	35.1 ± 4.5
16804543	spl	peptidoglycan lytic protein P45	0.0	0.0	0.0	0.8	4.3 ± 0.6			6.1 ± 1.0	10.1 ± 1.0	11.4 ± 2.9
16804414		peptidyl-prolyl cis-trans isomerase	15.8 ± 2.3	36.4 ± 13.0	19.9 ± 2.6	14.8 ± 0.3	14.7 ± 1.0	24.2 ± 3.8	24.2 ± 0.0	17.5 ± 5.7	22.8 ± 1.9	16.1 ± 3.8
16802177	ncaE	N-acetylmuramoyl-L-alanine amidase	2.5 ± 1.8	8.5 ± 3.6	9.9 ± 0.2	0.0	0.6			4.3 ± 0.0		
**DNA/RNA**												
16802093	ssb	single-strand binding protein	4.3 ± 1.2	0.0	6.6	0.0	0.0	5.9 ± 4.2	2.9			
16803973	hup	DNA-binding protein HU	1399.0 ± 4.2	392.4 ± 346.0	686.9 ± 3.8	2259.0 ± 57.1	2266.9 ± 49.9	1446.3 ± 65.7	839.3 ± 37.0	551.8 ± 8.2	897.4 ± 53.4	917.7 ± 0.0
기타												
16803619		alanine dehydrogenase	1.3 ± 0.6	3.8 ± 1.6	5.0 ± 0.1	3.1 ± 0.7	0.4	7.3 ± 2.1	9.4 ± 5.1	6.5 ± 1.0	1.5	3.6 ± 1.0
16803220		carboxysome structural protein	30.9 ± 2.4	10.5	7.0 ± 0.2	0.0	0.0	21.6 ± 4.4				
